# Novel hybrid action of GABA mediates inhibitory feedback in the mammalian retina

**DOI:** 10.1371/journal.pbio.3000200

**Published:** 2019-04-01

**Authors:** James C. R. Grove, Arlene A. Hirano, Janira de los Santos, Cyrus F. McHugh, Shashvat Purohit, Greg D. Field, Nicholas C. Brecha, Steven Barnes

**Affiliations:** 1 Department of Neurobiology, David Geffen School of Medicine, University of California, Los Angeles, California, United States of America; 2 Neuroscience Graduate Program, University of California, San Francisco, California, United States of America; 3 Veterans Administration Greater Los Angeles Healthcare System, Los Angeles, California, United States of America; 4 Doheny Eye Institute, University of California, Los Angeles, California, United States of America; 5 Department of Neurobiology, Duke University School of Medicine, Durham, North Carolina, United States of America; 6 Department of Medicine, David Geffen School of Medicine, University of California, Los Angeles, California, United States of America; 7 Department of Ophthalmology, David Geffen School of Medicine, University of California, Los Angeles, California, United States of America; 8 Stein Eye Institute, David Geffen School of Medicine, University of California, Los Angeles, California, United States of America; 9 Department of Physiology and Biophysics, Dalhousie University, Halifax, Nova Scotia, Canada; 10 Department of Ophthalmology and Visual Sciences, Dalhousie University, Halifax, Nova Scotia, Canada

## Abstract

The stream of visual information sent from photoreceptors to second-order bipolar cells is intercepted by laterally interacting horizontal cells that generate feedback to optimize and improve the efficiency of signal transmission. The mechanisms underlying the regulation of graded photoreceptor synaptic output in this nonspiking network have remained elusive. Here, we analyze with patch clamp recording the novel mechanisms by which horizontal cells control pH in the synaptic cleft to modulate photoreceptor neurotransmitter release. First, we show that mammalian horizontal cells respond to their own GABA release and that the results of this autaptic action affect cone voltage-gated Ca^2+^ channel (Ca_V_ channel) gating through changes in pH. As a proof-of-principle, we demonstrate that chemogenetic manipulation of horizontal cells with exogenous anion channel expression mimics GABA-mediated cone Ca_V_ channel inhibition. Activation of these GABA receptor anion channels can depolarize horizontal cells and increase cleft acidity via Na^+^/H^+^ exchanger (NHE) proton extrusion, which results in inhibition of cone Ca_V_ channels. This action is effectively counteracted when horizontal cells are sufficiently hyperpolarized by increased GABA receptor (GABAR)-mediated HCO_3_^−^ efflux, alkalinizing the cleft and disinhibiting cone Ca_V_ channels. This demonstrates how hybrid actions of GABA operate in parallel to effect voltage-dependent pH changes, a novel mechanism for regulating synaptic output.

## Abstract 2

Receptors for the neurotransmitter GABA in the dendritic tips of GABA-releasing horizontal cells in the retina mediate an autoreceptive mechanism that modulates presynaptic voltage-gated calcium channels in cone cells through changes in pH within the synaptic cleft.

## Introduction

Vision relies on reliable information transfer from photoreceptors to horizontal and bipolar cells at the first synapse in the visual system [1]. Horizontal cells integrate photoreceptor glutamate release and, in turn, modulate this release via inhibitory feedback [2,3]. The overall view is that by regulating the gain of photoreceptor output, horizontal cells help generate the receptive field properties of bipolar cells and ganglion cells [3–7]. However, our understanding of the cellular mechanisms that mediate this synaptic feedback circuit remains incomplete [2,3,8,9].

The voltage-gated Ca^2+^ channels (Ca_V_ channels) in the photoreceptor synaptic terminal are the known targets of horizontal cell feedback [10–15]. In nonmammalian vertebrates, horizontal cell release of GABA directly hyperpolarizes photoreceptors [16–21], but in mammalian retina, evidence for a GABA-activated chloride (Cl^−^) conductance in normal cones has not been established. Moreover, feedback inhibition to cones in fish [15] and macaque [14] is accompanied by a decrease in Ca^2+^ and Ca^2+^-activated Cl^−^ conductances, which is inconsistent with a direct ionotropic action on cones of GABA. Rather, these conductance changes are explained by decreased activation of cone Ca_V_ channels and their closely linked Ca^2+^-activated Cl^−^ channels [22,23], which are shown in this report to be indirectly modulated by GABA agonists and antagonists in a novel manner.

A widely supported model for feedback inhibition involves acidification of the photoreceptor synaptic cleft [3,24,25], with subsequent membrane surface charge effects [26] (e.g., proton interactions with the fixed negative surface charge of the bilayer and membrane protein–binding sites) that reduce photoreceptor Ca_V_ channel activation [27]. Many reports show that increased pH buffering with just 10 mM Hepes is enough to reversibly block horizontal cell feedback [2,10,11,13,28–30], implying that the HCO_3_ˉ/CO_2_ buffering system is neither open nor fast in this region of the retina [31].

Modest changes in synaptic cleft pH modulate the voltage dependence of photoreceptor Ca_V_ channel activation and powerfully alter glutamate release from photoreceptors [27], bolstering the evidence that activity-driven changes in pH in the synaptic cleft are responsible for synaptic regulation. While slow extracellular acidification normally accompanies neuronal depolarization due to the metabolic activity required to maintain ionic gradients [32], membrane mechanisms capable of rapid pH change, e.g., Na^+^/H^+^ exchangers (NHEs), Na^+^/HCO_3_^−^ cotransporters (NBCs), anion exchangers (AEs), Na^+^/Cl^−^/HCO_3_^−^ exchangers (NCBEs), and Na^+^-driven Cl^−^/HCO_3_^−^ exchangers (NDCBEs), vesicular ATPases (V-ATPases), monocarboxylic acid transporters (MCTs), and intra- and extracellular carbonic anhydrase enzymes (CAs) [33], are known to be or are likely present throughout the retina. Yet it remains unclear what exact adaptations have allowed for acidification to constitute this feedback mechanism.

Earlier, we proposed a model that includes a role for horizontal cell release of GABA [34–37]. In that scheme, GABA acts on horizontal cell GABA receptors (GABARs) autaptically [2,38–40], allowing the efflux of the permeant anion bicarbonate (HCO_3_ˉ) to buffer cleft pH [41,42], thereby modulating photoreceptor transmitter release via surface charge effects on presynaptic Ca_V_ channels [27]. The contribution of the GABAR channel to cleft pH would depend critically on the driving force on HCO_3_ˉ, which is a function of the equilibrium potential for HCO_3_ˉ (E_HCO3_^−^; typically in the range of −15 to −20 mV) and horizontal cell membrane potential, meaning that cone Ca_V_ channel disinhibition would be maximal at negative membrane potentials in which HCO_3_ˉ efflux is greatest. It remained unclear whether reduced HCO_3_ˉ efflux would be sufficient to drive inhibition of photoreceptor Ca_V_ channels at more positive horizontal cell voltages.

To elucidate the actions of GABA at the mammalian photoreceptor synapse, we utilized patch clamp techniques to directly measure feedback effects on photoreceptor Ca_V_ channel activation in three rodent species. Here, we show that HCO_3_^−^ efflux accounts for the disinhibition of photoreceptor Ca_V_ channels at hyperpolarized horizontal cell potentials and that reduction of the outward driving force on HCO_3_^−^ efflux and continuous proton extrusion by NHEs accounts for inhibition of photoreceptor Ca_V_ channels at depolarized horizontal cell potentials. We explain how these actions balance to mediate feedback to cones over the full range of horizontal cell membrane potential excursions. It is likely that other, mechanistically similar pH-mediated modulations of synaptic interaction occur throughout the brain and that what we show in this report is utilized for additional forms of regulation of excitability [43–46].

## Results

Experiments were performed in retinal slices from mice, rats, and guinea pigs, with replication of procedures in cone photoreceptors carried out in all three species in many cases to confirm quantitatively similar results (S1 Table). Horizontal cell types differ among these species, with guinea pigs having both A- and B-type horizontal cells, while mice and rats only have the B-type [47]. Differences in GABA synthesis between these species are also considered to be a result of varying levels of glutamic acid decarboxylase (GAD) [35,48]. In view of these differences, cross-species replication ensures greater confidence that any common effect represents a conserved mechanism.

The targets of horizontal cell inhibition in photoreceptors are the Ca_V_ channels, which are responsible for neurotransmitter release from their presynaptic terminals [10–15,29,49]. To determine the role of GABA in the inhibition of photoreceptor Ca_V_ channel activation, we performed our experiments under mesopic conditions, in which horizontal cells are relatively depolarized and GABA levels in outer plexiform layer (OPL) are relatively high. First, we tested whether a block of GABARs produces disinhibition of cone Ca_V_ channels.

### Ca_V_ channels in cone photoreceptors of three rodent species are maintained in a tonically inhibited state by GABAR activation

**Fig 1:**
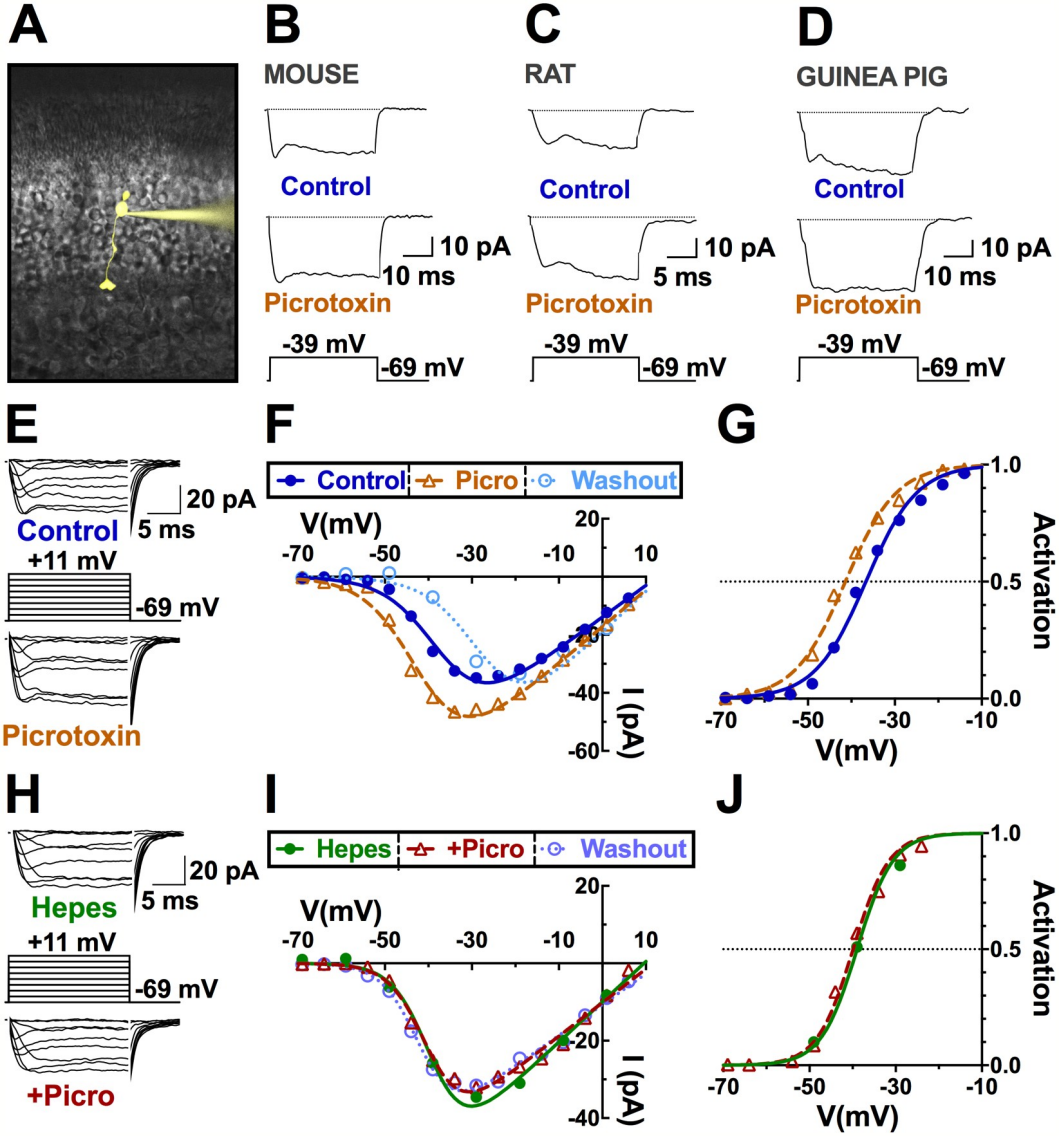
Ca_V_ channels in cone photoreceptors of mice, rats, and guinea pigs are maintained in a pH-mediated, tonically inhibited state by GABAR activation. A. Image of a mouse cone filled with Lucifer yellow via the patch clamp electrode. B–D. GABAR antagonist picrotoxin (100 μM) increases peak calcium current amplitude in mouse, rat, and guinea pig cones, maintained in mesopic conditions. Fine dashed lines indicate zero current. E,F. Sample current traces and I–V relations of a rat cone before, during, and after application of picrotoxin. G. The Ca_V_ channel activation curve for the cell in (E) reveals a leftward shift in the V_½_ in picrotoxin (V_½_ = −41 mV) versus control (V_½_ = −37 mV). H–J. Same experiment in E–G in the same cell while clamping pH to 7.4 with the pH buffer Hepes (10 mM), eliminating the effect of picrotoxin on Ca_V_ channel activation (V_½_ = −39 mV). This implies a tonic GABA inhibition that is pH dependent. Underlying data of cells in this figure can be found in S1 Data. Ca_V_ channel voltage-gated Ca^2+^ channel; I–V, current–voltage; V_½_, half-maximal activation voltage.

Using the whole-cell patch technique to voltage clamp the Ca_V_ channel currents of cones in retinal slices (Fig 1A), we found that 100 μM picrotoxin, an antagonist of most GABARs, increased peak Ca_V_ channel current in cones by 57% ± 20% in mice (Fig 1B; *n* = 5), 38% ± 16% in rats (Fig 1C; *n* = 5), and 24% ± 8% in guinea pigs (Fig 1D; *n* = 7). Measured under the voltage clamp paradigm shown in Fig 1E, the current–voltage relations of peak Ca_V_ channel current before, during, and after 100 μM picrotoxin application (e.g., in rat, Fig 1F) were divided by the driving force for calcium and fit with a Boltzmann function to derive Ca_V_ channel activation curves. The half-maximal activation voltage (V_½_) shifted leftward (to a more negative voltage) by 5.5 ± 0.8 mV in rat cones (Fig 1G; CI: 3.9–6.8; *P* < 0.001; *n* = 5), by 5.6 ± 1.2 mV (CI: 3.4–7.6; *P* < 0.001; *n* = 5) in mouse cones, and by 4.6 ± 1.0 mV (CI: 2.8–6.4; *P* < 0.001; *n* = 5) in guinea pig cones. This leftward shift of the channel activation curve to more negative potentials in the presence of picrotoxin effectively disinhibits cone Ca_V_ channel currents by increasing channel open probability at physiological membrane potentials. These results imply a tonic GABA inhibition of cone Ca_V_ channels under mesopic conditions in these species.

### Tonic inhibition of cone Ca_V_ channels reflects pH-mediated modulation of the voltage dependence of channel gating

These findings are consistent with previous reports that horizontal cells produce their inhibitory actions on photoreceptors by shifting the photoreceptor Ca_V_ channel activation curve in a pH-dependent manner [11,29]. In the Hepes-containing solution (10 mM; pH 7.4), this negative shift was eliminated. Fig 1H–1J shows that adding Hepes to the bath effectively abolished the effect of picrotoxin (*P* = 0.13; *n* = 5; comparison to control: *P* < 0.001), indicating that picrotoxin’s effect was pH dependent. Similar effects were seen in mouse and guinea pig (see S1 Data). The disinhibition evoked by picrotoxin here is the opposite of the effect seen using Ca^2+^ imaging [2], which was recorded under saturating photopic conditions with horizontal cells maximally hyperpolarized.

Picrotoxin, which blocks the ion channel pore of ionotropic GABA_A_ receptors, including some but not all of those containing rho (ρ) subunits [50] as well as glycine receptors [51] did not decrease standing-cone conductance measured between −80 and −50 mV. There was no difference in cone conductance with and without picrotoxin in mice (*P* = 0.7, *n* = 5), rats (*P* = 0.9, *n* = 5), or guinea pigs (*P* = 0.15, *n* = 7; S2 Table). This suggests that there is no tonic, direct GABAergic input onto photoreceptor terminals.

While these results support previous findings showing the pH sensitivity of inhibitory horizontal cell feedback to photoreceptor Ca_V_ channels [10,11,13,25,29,52] and previous reports in mammals regarding the actions of GABA antagonists in rat [2] and macaque [14], they do not identify the inhibitory amino acid receptor responsible for the disinhibitory action of picrotoxin.

### GABA receptors containing ρ-subunits mediate the modulation of cone Ca_V_ channel currents

**Fig 2:**
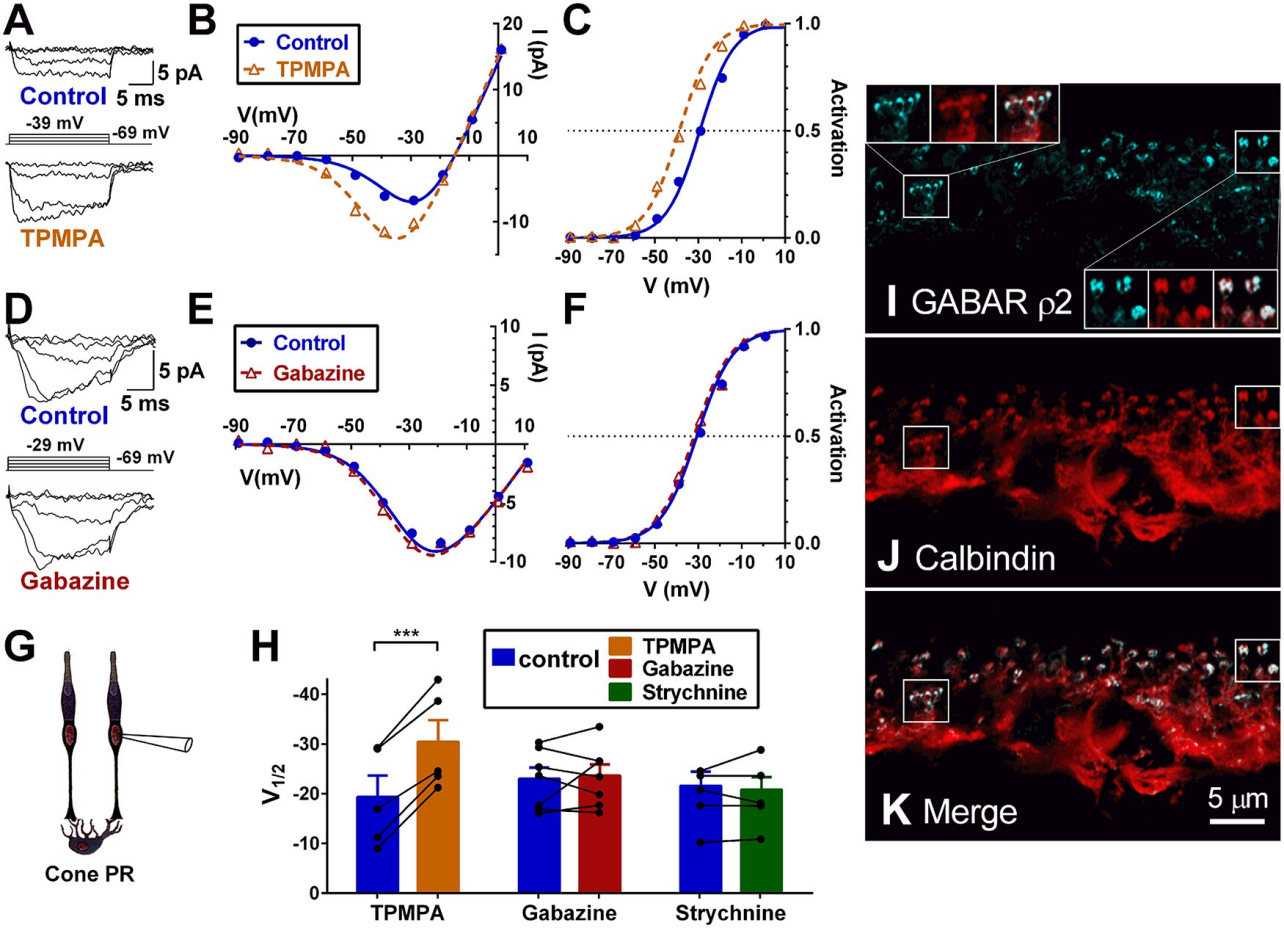
GABARs containing ρ-subunits mediate the modulation of cone Ca_V_ channel currents, and ρ-subunits are expressed in horizontal cell synaptic tips. Effects of GABAR blockers on Ca_V_ channel currents in cones in guinea pig retinal slices. A–C. The ρ-subunit–containing GABAR antagonist TPMPA (50 μM) shifts Ca_V_ channel activation in guinea pig cones to more negative voltages (V_½_ = −29 versus V_½_ = −39 for cell shown). D–F. The GABA_A_R antagonist gabazine (10 μM) has no effect on Ca_V_ channel activation. G. Graphic to show all recordings in the figure are made from cones. H. Summary showing the effects of TPMPA (*n* = 5), gabazine (*n* = 7), and the GlyR antagonist strychnine (100 μM; *n* = 6) on Ca_V_ channel activation. I. GABAR ρ2 subunit (blue) and calbindin (red; J) immunoreactivity in mouse retina with maximum intensity projections. The ρ2 subunit is strongly expressed in the tips of the horizontal cell processes (merged in K and enlarged insets in I: cone pedicle, upper left; rod spherules, lower right), where they enter the photoreceptor terminals. Underlying data of cells in this figure can be found in S1 Data. Ca_V_ channel, voltage-gated Ca^2+^ channel; GABAR, GABA receptor; GlyR, glycine receptor; TPMPA, (1,2,5,6-tetrahydropyridin-4-yl)methylphosphinic acid.

**Fig 3:**
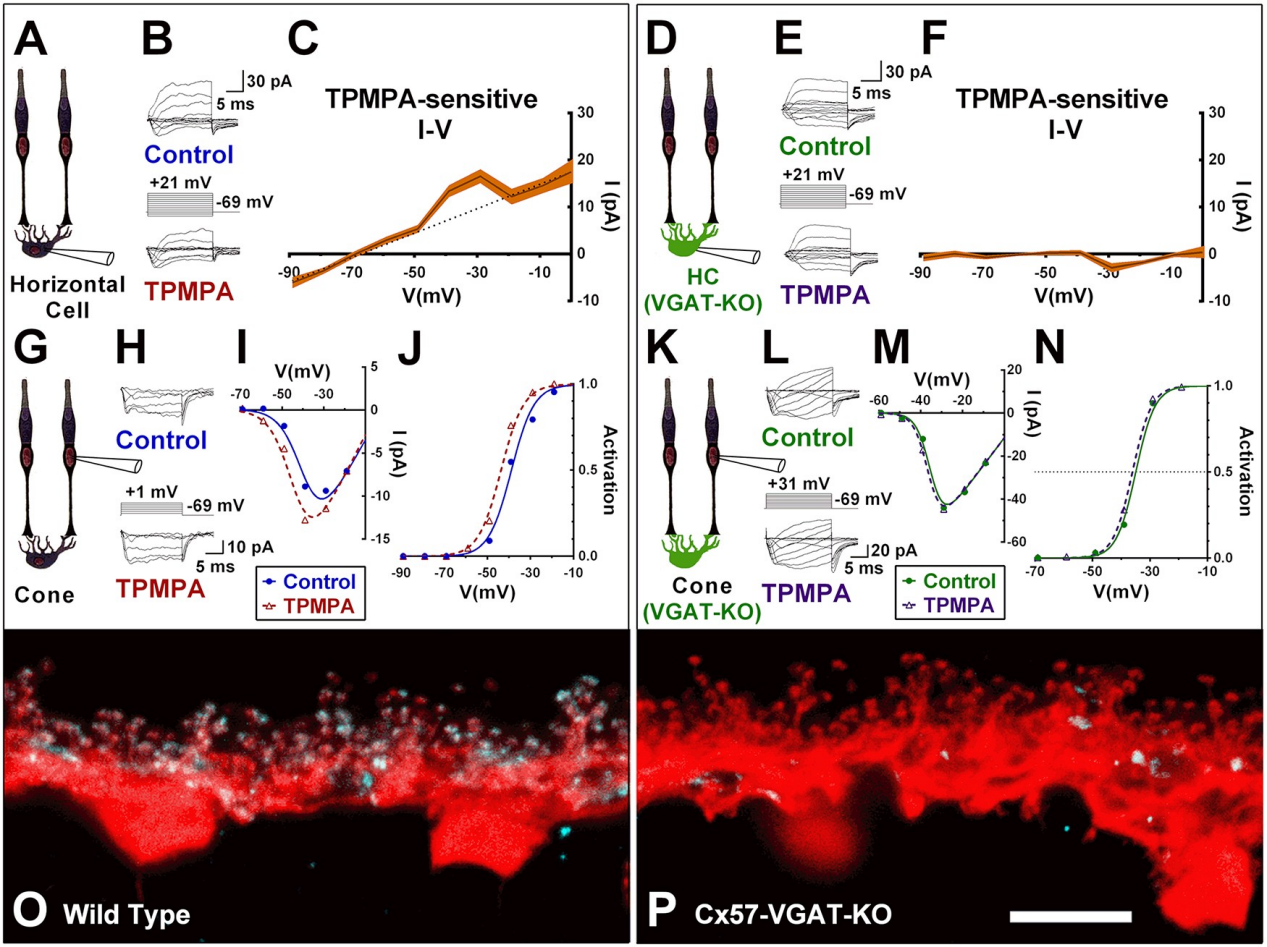
Tonic horizontal cell release of GABA activates ρ-subunit containing GABARs on its own cellular membrane, which indirectly inhibits cone Ca_V_ channels. A. Patch clamp recording of a tdTomato labeled mouse horizontal cell in a slice. B. Currents elicited in a horizontal cell bathed in 50 μM CNQX and 10 mM Hepes by voltage steps in control (top) and in 50 μM TPMPA (below). C. Average I–V relation of TPMPA-subtracted current for five cells shows a linear component reversing near −67 mV (dotted line is linear fit excluding values at −39 and −29 mV). Width of orange line shows the standard deviation for the 5 cells. D–F. Same experiment as in A-C but in Cx57-VGAT-KO mouse horizontal cells [37]. Mouse horizontal cells lacking VGAT are unaffected by TPMPA. G. Patch clamp recording of cone in wild-type mouse. H. Current in the absence and presence of TPMPA. I–V relations (I) and activation curves (J) of the cell in (H) shows that the activation midpoint shifted from −39 mV to −44 mV with TPMPA application. K–N. Same experiment in A–C in a cone of a Cx57-VGAT-KO mouse. Unlike cones in wild-type retinas, the activation midpoint is unaffected by TPMPA in the VGAT-deleted cones. O–P. VGAT (blue) and calbindin (red) immunoreactivity in wild-type (O) and Cx57-VGAT-KO (P) mouse retinas (scale bar = 10 μm). Sparse labeling for VGAT in (P) represents projections from cells in the inner retina. The super-resolution confocal image (maximum intensity projection) in O shows VGAT immunolabeling in horizontal cell endings that correspond to the same cellular compartment the GABAR ρ2 subunits were seen localized in Fig 2I–2K. Underlying data of cells in this figure can be found in S1 Data. Ca_V_ channel, voltage-gated Ca^2+^ channel; CNQX, 6-cyano-7-nitroquinoxaline-2,3-dione; GABAR, GABA receptor; I–V, current–voltage; KO, knockout; TPMPA, (1,2,5,6-tetrahydropyridin-4-yl)methylphosphinic acid; VGAT, vesicular GABA transporter.

To determine which GABAR subtype is responsible for the disinhibition of Ca_V_ channels in guinea pig cones, we compared the Ca_V_ channel activation curve shift induced by the ρ-subunit containing GABAR inhibitor (1,2,5,6-tetrahydropyridin-4-yl)methylphosphinic acid (TPMPA), the GABA_A_R inhibitor gabazine, and the glycine receptor inhibitor strychnine. Superfusion with the GABA_A_ ρ-subunit receptor antagonist TPMPA (50 μM) increased Ca_V_ channel current amplitude, with an associated negative shift of the Ca_V_ channel activation curve, whereby V_½_ decreased by 11.1 ± 1.1 mV (Fig 2A–2C; CI: 9.0–12.9; *P* < 0.001; *n* = 5). TPMPA had a similar effect in mice, shifting the activation curve V_½_ leftward by 5.7 ± 0.7 mV (Fig 3H–3J; CI: 4.2–6.8; *P* = 0.0006 < 0.001). Gabazine (10 μM) and strychnine (100 μM) did not cause similar activation curve shifts (gabazine: Fig 2D–2F, *P* = 0.69, *n* = 7; strychnine: *P* = 0.56, *n* = 6). Data for all three antagonists are summarized in Fig 2H. These results indicate that ρ-subunit–containing GABARs mediate the inhibitory effects of GABA on cone presynaptic Ca_V_ channels.

Similar to the result with picrotoxin, TPMPA, gabazine, and strychnine did not produce conductance decreases in cones between −80 and −50 mV (TPMPA: *P* = 0.4, *n* = 5; gabazine: *P* = 0.1, *n* = 7; strychnine: *P* = 0.4, *n* = 6), suggesting that GABARs are absent from cones (S2 Table).

Due to the absence of evidence for mammalian photoreceptor GABARs and reports of GABARs on horizontal cells and bipolar cells [2,53], we hypothesized that horizontal cells might be the primary and possibly exclusive site of action for GABA in this feedback. There are reports of ρ-subunit–containing GABAR-mediated responses in fish and salamander horizontal cells [39,54–58] as well as in mouse horizontal cells [2,40]. We turned to immunohistochemistry to determine the site of action of GABA in the outer retina feedback circuit.

### GABARs containing ρ-subunits are expressed in horizontal cell synaptic tips

GABA and the GABAR agonist muscimol activate ionotropic GABAR channels on acutely isolated mouse horizontal cells [2,40,59]. Super-resolution confocal images of immunostained retinal sections show colocalized expression of GABAR ρ2 subunits with the horizontal cell marker calbindin in horizontal cell synaptic tips (Fig 2I–2K). These images show the characteristic apposing lateral elements typical of electron microscopic images of photoreceptor invaginating synapses. While additional GABAR α_1_ and γ_2_ subunits are reported to be localized to other horizontal cell compartments [53], ρ-subunit–containing GABARs appear to be specifically localized on or near the tips of horizontal cell dendritic and axonal processes.

If ρ-subunit–containing GABARs are present on these tips, we should be able to detect currents that are sensitive to antagonists of these receptors in horizontal cells. We next patch clamped horizontal cells in slices and applied TPMPA to test for the presence of currents due to ρ-subunit–containing GABARs that were tonically activated in horizontal cells.

### Autaptic action of GABA in horizontal cells: Tonic, depolarization-induced current blocked by TPMPA

Switching the recorded cell type from cones to horizontal cells, Fig 3A–3C shows the results from Cx57-tdTomato–labeled mouse horizontal cells voltage clamped to steps between −80 and +21 mV in slices without and then with TPMPA (50 μM; Fig 3B). The two sets of currents were digitally subtracted (control current minus current in the presence of TPMPA, i.e., TPMPA insensitive current), isolating a mostly linear TPMPA-sensitive current (Fig 3C), reversing at −67 ± 1.6 mV with a conductance of 0.26 ± 0.03 nS (*n* = 5). The linear current component, blocked by TPMPA, is consistent with the presence of a standing GABA-activated Cl^−^ current in the horizontal cell. Note that a portion of the current blocked by TPMPA was supralinear at the approximate values of peak Ca_V_ channel current in horizontal cells (e.g., −30 and −40 mV; Fig 3C; *P* < 0.01; *n* = 5). While not having ruled out rundown of Ca_V_ channel current as causing this, we speculate that it could reflect TPMPA blocking an additional, calcium-stimulated autaptic Cl^−^ current produced by horizontal cells releasing GABA at those specific voltages, activating their own GABARs.

Since these experiments were performed in 6-cyano-7-nitroquinoxaline-2,3-dione (CNQX) (50 μM) with the bath buffered, with 10 mM Hepes added at constant pH (7.4) to eliminate influences from photoreceptor-released glutamate and possible pH changes occurring in the synaptic cleft, all other horizontal cells should be hyperpolarized with little activated Ca_V_ channel current. The presence of a tonic current that can be blocked with TPMPA suggests that GABA is tonically released or not easily removed from the synaptic cleft or both. Abundant evidence suggests that horizontal cells release GABA, but GABA might also be released by interplexiform cells or diffuse to the OPL by bulk flow from inner retinal cells [60]. To reveal whether synaptic cleft GABA is a result of horizontal cell release, we recorded from horizontal cells in retina in which the vesicular GABA transporter (VGAT) that loads vesicles with GABA was specifically deleted in horizontal cells (Fig 3O and 3P) [37]. The horizontal cells in these mice are incapable of releasing GABA [61].

When we recorded from horizontal cells lacking VGAT (Cx57-VGAT-KO; Fig 3D), we found TPMPA no longer caused any change in current (Fig 3E and 3F; *P* = 0.8, *n* = 5), differing significantly from wild-type (WT) mice (*P* < 0.001), despite the normal expression of ρ-subunit–containing GABARs in horizontal cells of Cx57-VGAT-KO animals (S1 Fig). These negative findings with the Cx57-VGAT-KO mice, alongside the observation of increased outward current around peak calcium current in the WT, suggest that horizontal cells autaptically respond to the GABA they release.

### Actions of TPMPA on cone Ca_V_ channels depend on release of GABA from horizontal cells

The experiments described above show that GABA autoreception by horizontal cells is eliminated in the Cx57-VGAT-KO mice. To test if the cone Ca_V_ channel currents are still modulated, we recorded these currents in cones from Cx57-VGAT-KO mice. While the experiments in Fig 3G–3J confirmed that TPMPA causes negative shifts in cone Ca_V_ channel activation midpoint in WT mice (summarized above), results shown in Fig 3K–3N reveal that TPMPA failed to cause a significant shift in cone Ca_V_ channel activation in Cx57-VGAT-KO mice (ΔV_1/2_ = −3.3 ± 2.7 mV; CI: −8.2–1.2; *P* = 0.16; *n* = 5; comparison to WT: *P* < 0.001). Thus, recordings obtained from horizontal cells and cones of the Cx57-VGAT-KO animals suggest that GABA, released by horizontal cells and acting locally on horizontal cells, is responsible for the Ca_V_ channel activation curve shifts in cones (Fig 3J versus 3N). Since these results do not discriminate between whether the activation of GABAR channels on horizontal cell tips causes cone Ca_V_ channel inhibition or whether the two phenomena are merely correlated, we used a chemogenetic tool to test the link between the two actions.

### Cone Ca_V_ channel inhibition can be reproduced with an engineered anion channel in horizontal cells

**Fig 4:**
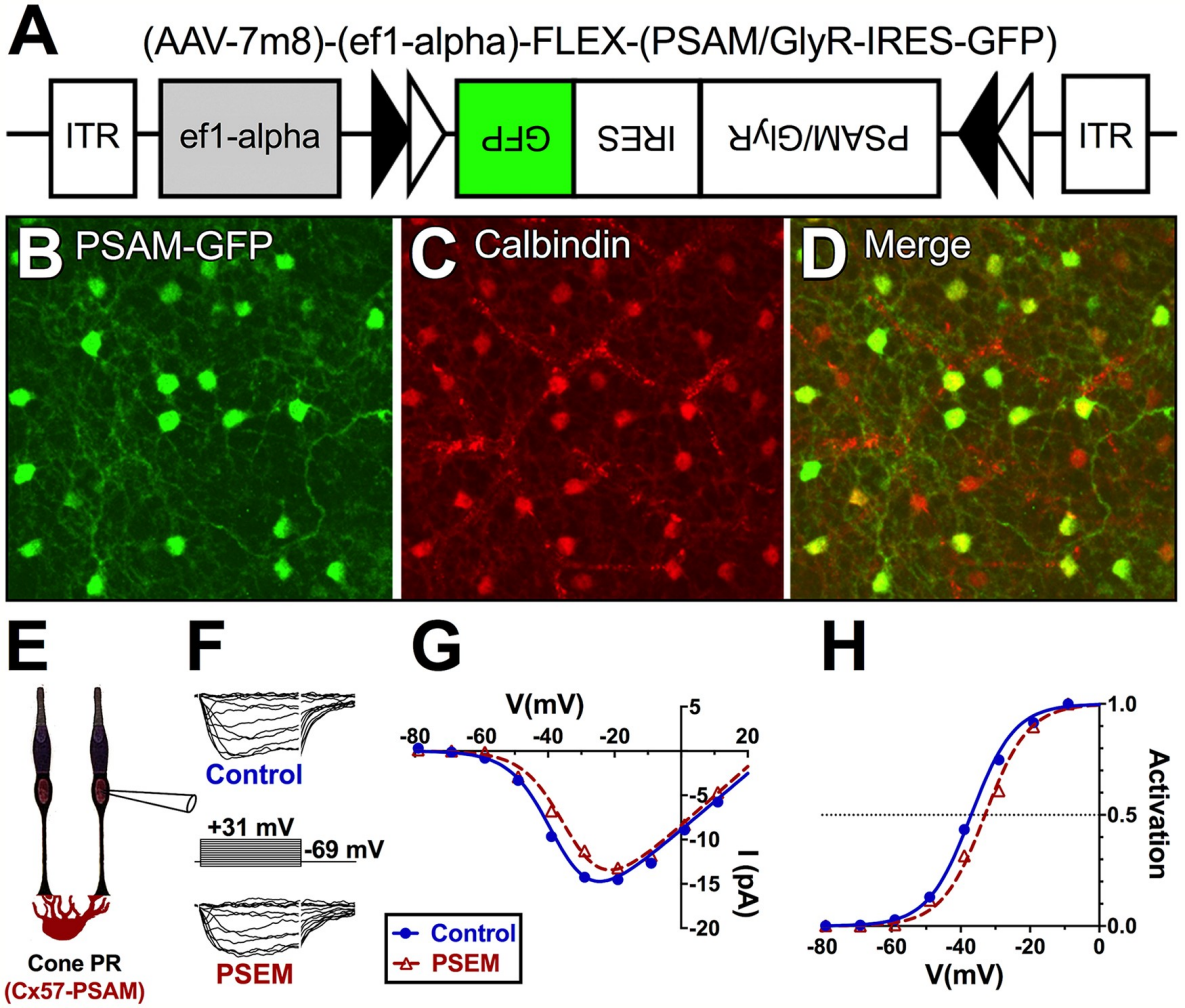
Activation of exogenously expressed Cl^−^/HCO_3_^−^ channels on horizontal cell membranes is sufficient to inhibit Ca_V_ channels in mouse cones. A. AAV-7m8-PSAM-GlyR-IRES-GFP construct. Cre-expressing horizontal cells transduced with this virus express Cl^−^/HCO_3_^−^ permeable GlyR complexes that can be activated by the orthogonal ligand PSEM. B. GFP fluorescent somata (reporter for AAV-7m8-PSAM-GlyR). C. Calbindin immunostaining of horizontal cell bodies in a whole-mount retina. D. Merged image focused on the distal INL. Most calbindin-labeled cell bodies express GFP, and nonspecific staining of blood vessels is visible. E. Patch clamp recording of a cone of a Cx57-PSAM-GlyR mouse. F. Currents elicited by the voltage steps shown in the absence (top) and presence (bottom) of 200 nM PSEM. I–V plots (G) and activation curves (H) of the cell in (F) reveal a rightward shift of activation midpoint with PSEM application, in a manner similar to the GABA agonist muscimol in the following figure. Underlying data of cells in this figure can be found in S1 Data. AAV, adeno-associated virus; GFP, green fluorescent protein; GlyR, glycine receptor; INL, inner nuclear layer; IRES, internal ribosome entry site; I–V, current–voltage; PSAM, pharmacologically selective actuator module; PSEM, pharmacologically selective effector molecule.

To examine whether activation of a Cl^−^/HCO_3_^−^ conductance having properties similar to GABAR channels on horizontal cells can modulate inhibition of Ca_V_ channels in cones, we engineered a Cre-dependent viral construct using adeno-associated virus (AAV)-7m8 [62] to transduce Cx57-iCre-expressing mouse horizontal cells [61] with a pharmacologically selective actuator module (PSAM) construct (Fig 4A–4D), an engineered glycine receptor (GlyR) channel activated by application of the orthogonal ligand called pharmacologically selective effector molecule (PSEM^308^) at concentrations as low as 200 nM [63]. PSEM^308^ is not known to bind to any naturally occurring receptor at 200 nM [64]. We refer to these transduced mice, whose horizontal cells express green fluorescent protein (GFP), as Cx57-PSAM-GlyR mice.

Like the glycine receptor the engineered channel is based on, PSAM-GlyR channels are permeable to Cl^−^ and HCO_3_^−^. We confirmed the Cl^−^ permeability of this channel in the transduced horizontal cells with cesium chloride (CsCl)- and K^+^-gluconate–based internal solutions (S2 Fig). If GABAR-mediated inhibition of cone Ca_V_ channel current depends on the flux of either of these ions across the horizontal cell membrane, PSEM^308^, which activates an anionic conductance, should inhibit cone Ca_V_ channels in Cx57-PSAM-GlyR mice.

Fig 4F shows Ca_V_ channel currents elicited from cones in Cx57-PSAM-GlyR mice by the voltage clamp steps in the absence (top) and presence (bottom) of 200 nM PSEM^308^. Ca_V_ channel currents became smaller in the presence of PSEM^308^ as cone Ca_V_ channel activation curve shifted rightward by 7.5 ± 1.9 mV (Fig 4H; CI: 11.2–4.1; *P* < 0.001; *n* = 8). PSEM^308^ did not have any effect on cone Ca_V_ channel activation in nontransduced retinas (*P* = 0.78; *n* = 3; see S1 Data; comparison to infected: *P* < 0.001), ruling out off-target effects influencing the results of these experiments.

This finding implicates the flux of either Cl^−^ or HCO_3_^−^ across the horizontal cell membrane in the GABA-mediated inhibition of cone Ca_V_ channels. Next, we sought to discriminate the roles the two anions might have in this process.

### GABAR-mediated inhibition of cone Ca_V_ channels depends on horizontal cell depolarization, mediated by [Cl^−^]

Low pH-mediated GABAergic inhibition of Ca_V_ channels cannot be simply attributed to bicarbonate flowing into horizontal cells because the outward rectification provided by big Ca^2+^-activated K^+^ (BK) channels [65] prevents horizontal cell depolarization positive to E_HCO3_^−^, which is typically in the range of −15 to −20 mV. Any effect of bicarbonate under the mesopic conditions in which we recorded, with horizontal cells at a resting membrane potential that is negative to E_HCO3_^−^ ought to be disinhibitory, as bicarbonate would flow out of horizontal cells and into the synaptic cleft. We therefore examined the role of chloride.

**Fig 5:**
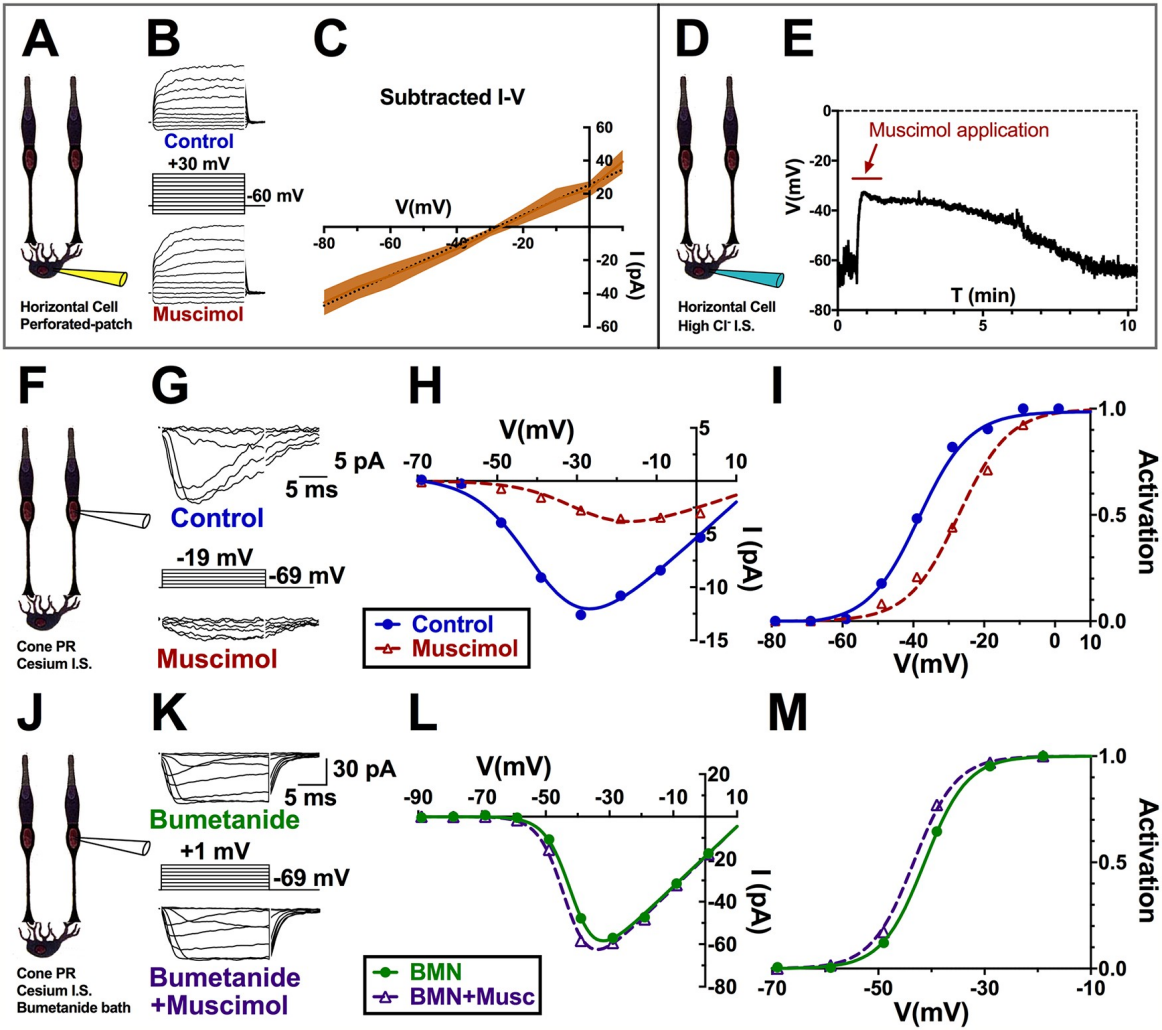
GABAR inhibition of cone Ca_V_ channels depends on horizontal cell [Cl^−^]_i_. A. Gramicidin-perforated patch clamp recordings of tdTomato-labeled mouse horizontal cells. B. Currents elicited by the voltage steps shown in control (top) and in the presence of 100 μM muscimol. C. Average I–V relations of muscimol-subtracted current (*n* = 5) shows the principle linear component reversing at −28 mV (dotted line is linear fit of mean subtracted currents). D. Whole-cell patch clamp recording of a mouse horizontal cell with a K^+^-gluconate-based internal solution containing 41 mM chloride. E. Time course of membrane potential, recorded under current-clamp, during response to muscimol (approximately 1 min application). F. Patch clamp recording of a mouse cone. G. Currents elicited in mouse cones by the voltage steps shown in the absence (top) and presence (bottom) of muscimol. H. I–V relations show smaller calcium currents in the presence of muscimol. I. Activation curves of the cell in (G) reveal a rightward V_½_ shift in muscimol. J–M. Same experimental paradigm as in F–I in mouse cones bathed in the NKCC blocker bumetanide. K. Currents in the absence and presence of muscimol with bumetanide pretreatment (30 min). I–V curves (L) and activation curves (M) of the cell in (K) reveal a slight leftward V_½_ shift with muscimol in bumetanide-treated retinas. Underlying data of cells in this figure can be found in S1 Data. Ca_V_ channel; voltage-gated Ca^2+^ channel; GABAR, GABA receptor; I–V, current–voltage; NKCC, Na^+^/K^+^/Cl^-^ cotransporter; V_½_, half-maximal activation voltage.

In gramicidin-perforated patch recordings of mouse horizontal cells with voltage steps from −80 to +30 mV in 10 mV increments, Fig 5A–5C shows that the GABAR agonist muscimol (100 μM) elicits a large current reversing near −28 ± 3 mV (*n* = 5). Gramicidin-perforated patch recording preserves physiological intracellular chloride levels, allowing us to determine the level of chloride in horizontal cells based on a P_HCO3_/P_Cl_ of 0.18 for horizontal cell GABAR channels [2]. The positive E_Cl_ suggests a greater intracellular chloride concentration (approximately 41 mM) in horizontal cells than is typical for mature neurons, as previously reported [66]. Voltage clamp recordings with Kgluconate-based internal solution containing 41 mM chloride in Fig 5E shows that muscimol depolarizes horizontal cells under these conditions from −56.8 ± 10.2 mV (*n* = 7) to −38.7 ± 0.9 mV (*n* = 5).

Previous studies reported the presence of the chloride transporter NKCC1 in horizontal cells, but not in cones [67]. NKCC1 (Slc12a2) is a Na^+^/K^+^/Cl^−^ cotransporter that moves Cl^−^ into cells, producing a relatively positive equilibrium potential for Cl^−^ [68,69]. The activity of NKCC1 causes neurons to accumulate high chloride concentrations and makes chloride channel activation depolarizing. If GABAergic inhibition of Ca_V_ channel in cones is due to the depolarizing effects of chloride, then block of NKCC1 should eliminate this effect.

Bumetanide, which blocks NKCC1 in neurons and reduces intracellular [Cl^−^] [70], changed the sign of cone Ca_V_ channel modulation by muscimol. In mouse retinas recorded under control conditions, shown in Fig 5F–5I, 100 μM muscimol produced a rightward shift in the Ca_V_ channel current activation curve by 7.4 ± 2.3 mV (CI: 11.2–3.3; *P* < 0.01; *n* = 4). In retinas pretreated with 50 μM bumetanide, however, 100 μM muscimol caused the Ca_V_ channel current activation curve midpoint to shift leftward by 2.0 ± 0.35 mV (Fig 5J–5M; CI: 1.4–2.7; *P* < 0.001; *n* = 5). This reduced shift-of-activation by muscimol in bumetanide was significantly different from the pronounced shift caused by muscimol in control (*P* < 0.001).

The disinhibitory effect of muscimol in the presence of bumetanide would be consistent with a block of NKCC1 in horizontal cells, leading to a drop in intracellular [Cl^−^] to low enough levels that GABAR activation no longer depolarizes them and may even produce hyperpolarization. It would follow that the removal of the depolarizing effect of GABA allows other actions, such as increased HCO_3_^−^ efflux, to dominate and alkalinize the synaptic cleft.

With evidence that the inhibitory effect of autaptically released GABA on cone Ca_V_ channel current is due to its depolarizing effect on horizontal cells, we sought to identify the role of cleft-acidifying processes that are initiated or increased when horizontal cells depolarize.

### NHEs mediate the cleft acidification underlying GABAR inhibition of cone Ca_V_ channels

NHEs have been implicated in horizontal cell–mediated feedback inhibition of photoreceptors in nonmammalian vertebrates [52]. Although the electroneutral H^+^ extruder has no intrinsic voltage sensitivity, its activity is greater in depolarized neurons due to the need for proton extrusion in metabolically active neurons and to its sensitivity to intracellular pH (pH_i_) and internal calcium levels, which increases with depolarization [71–75]. To investigate the role of this exchanger in mammalian horizontal cell feedback, we tested whether the inhibitory effect of GABA on cone Ca_V_ channels might be associated with increased NHE activity.

**Fig 6:**
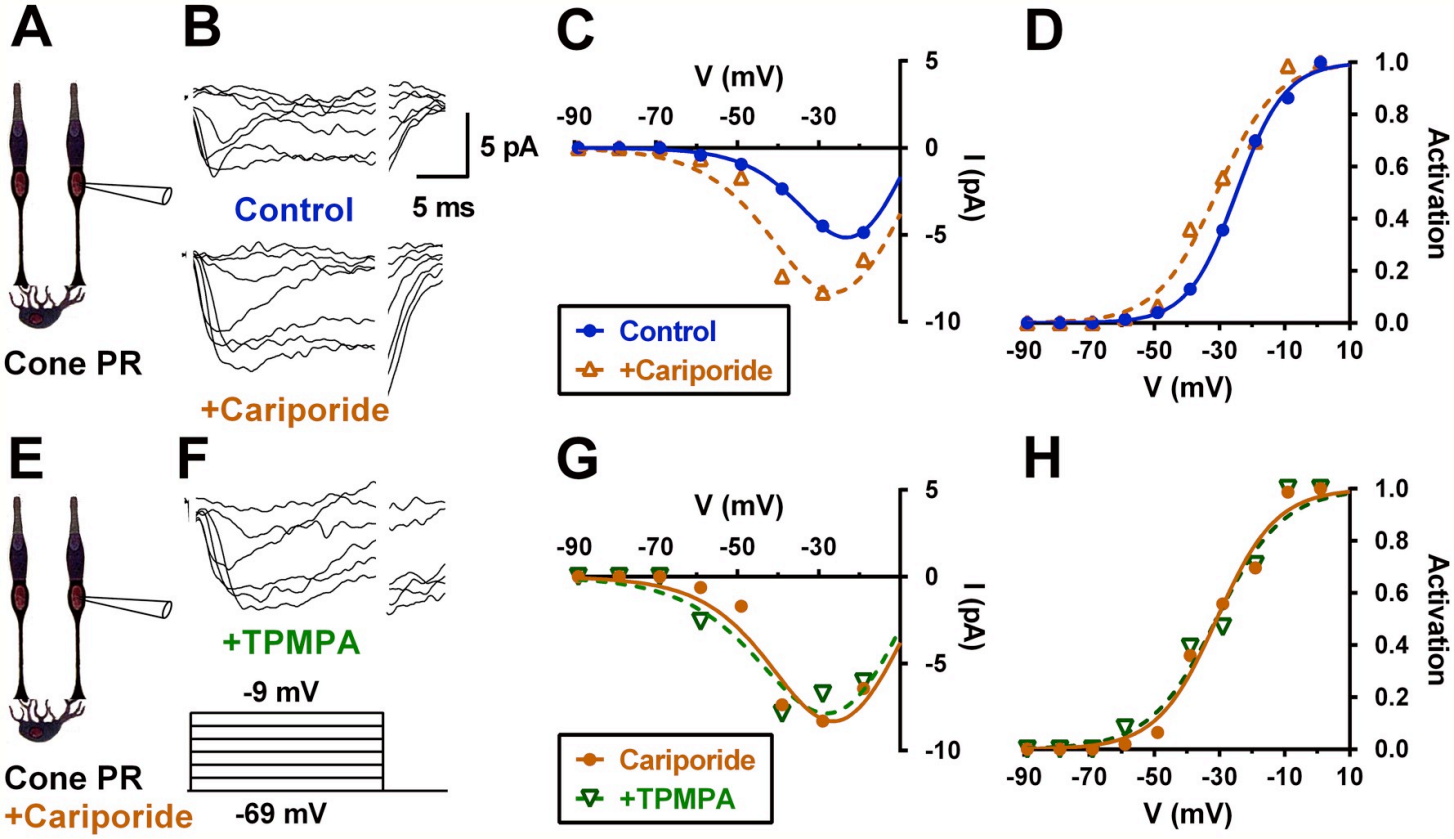
Blocking Na^+^/H^+^ exchangers with the selective inhibitor cariporide disinhibits cone Ca_V_ channels and eliminates the disinhibitory effect of TPMPA. A. Patch clamp recording of a mouse cone. B. Currents elicited by voltage steps shown in a mouse cone bathed in control bath alone (top), after adding 10 μM cariporide (middle), and after adding 50 μM TPMPA with the cariporide (bottom). C. I–V relations show larger Ca_V_ channel currents in the presence of cariporide, similar to the effects of the GABA_C_R antagonist TPMPA (cf. Fig 3G–3J). D. The cone Ca_V_ channel activation curve shifts to a more negative potential during cariporide application, from −15.8 mV to −21.9 mV. E–G. I–V relations show little effect of TPMPA on Ca_V_ channel current in the same mouse cone now bathed continuously with cariporide. H. In the presence of cariporide, TPMPA fails to shift Ca_V_ channel activation curve to more negative potentials (−21.9 mV to −21.9 mV). Underlying data of cells in this figure can be found in S1 Data. Ca_V_ channel, voltage-gated Ca^2+^ channel; GABAR, GABA receptor; I–V, current–voltage; TPMPA, (1,2,5,6-tetrahydropyridin-4-yl)methylphosphinic acid.

Application of the selective NHE-blocker cariporide (10 μM) shifted mouse cone Ca_V_ channel activation curves negative by 5.8 ± 1.24 mV (CI: 3.7–8.0; *P* < 0.001; *n* = 8; Fig 6A–6D), consistent with NHE having had an acidifying effect on the photoreceptor synaptic cleft at rest. These actions are comparable to the 5.7 ± 0.71 mV leftward shift produced by the GABAR antagonist TPMPA (see Fig 2). But in the presence of 10 μM cariporide (Fig 6F–6H), the GABAR antagonist TPMPA (50 μM) no longer shifted the Ca_V_ channel activation curve (−1.4 ± 1.7 mV; CI: 4.5– −1.7; *P* = 0.4; comparison to control: *P* < 0.001; *n* = 5).

Amiloride (30 μM), another blocker of NHE, produced similar results, shifting cone Ca_V_ channel activation negative by 4.9 ± 0.8 mV (CI: 3.4–6.2; *P* < 0.001; *n* = 5; S3 Fig). In 10 μM amiloride, TPMPA produced a slight rightward shift in the activation curve of cone Ca_V_ channels (0.8 ± 0.4 mV; CI: 1.5–0.2; *P* < 0.01; *n* = 5; comparison to control: *P* < 0.001; S3 Fig). This suggests that the inhibitory effects of GABARs are due at least in part to conditions affected by NHE activity.

These findings provide evidence that autaptically released GABA inhibits cone Ca_V_ channels in mesopic conditions by depolarizing horizontal cells and allowing increased NHE activity to dominate, lowering the cleft pH. However, GABA might not have the same effect at all horizontal cell potentials. In our previous study, GABA increased rat photoreceptor Ca_V_ channel currents under strong photopic conditions [76]. GABA may therefore have alkalinizing effects when horizontal cells are hyperpolarized.

### The polarity of cone Ca_V_ channel modulation depends on the horizontal cell membrane potential

**Fig 7:**
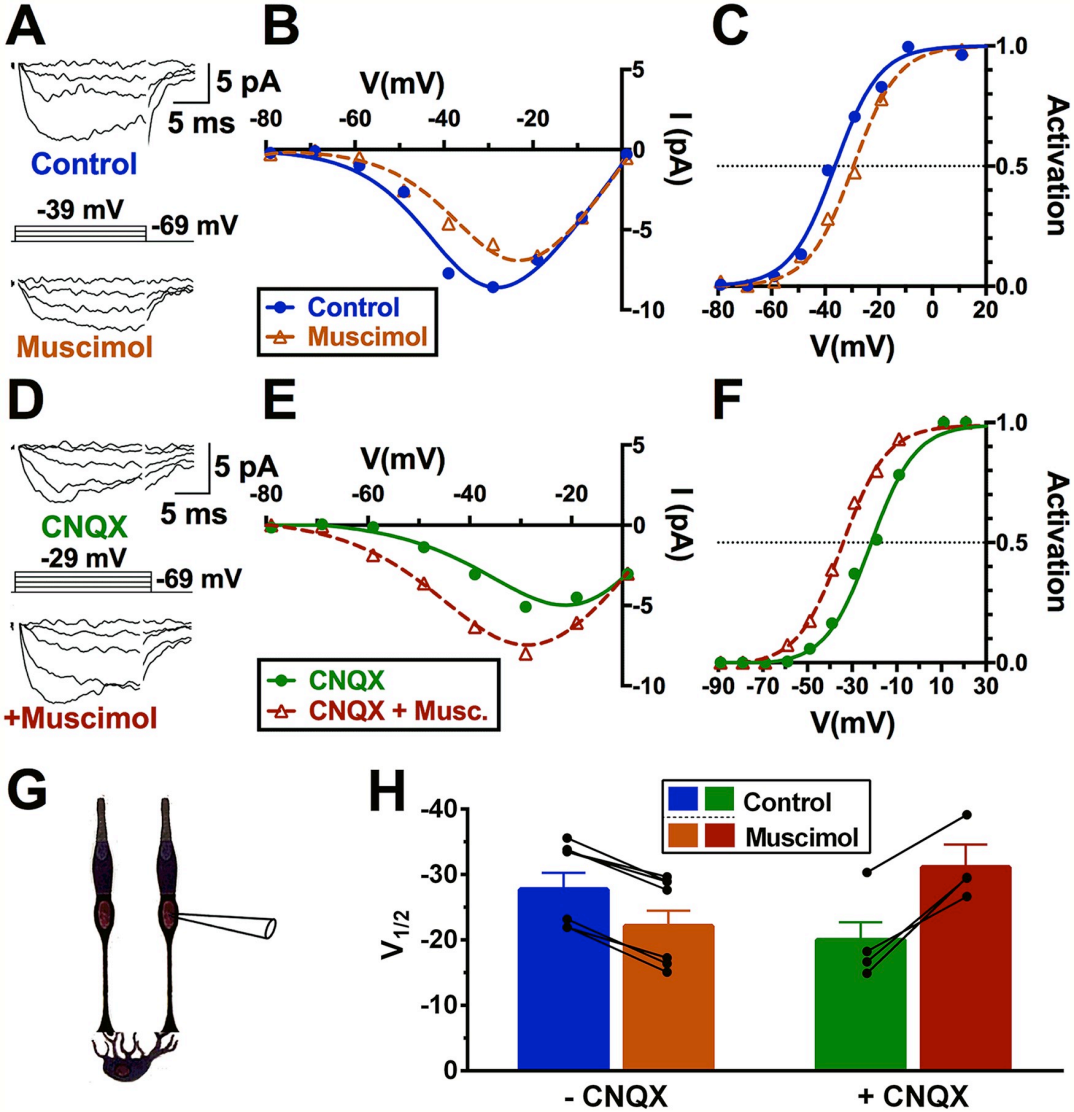
Polarity of cone Ca_V_ channel modulation depends on horizontal cell membrane potential. A. Currents elicited by the voltage steps shown in the absence (top) and presence (bottom) of 100 μM muscimol during whole-cell patch clamp recordings of a cone in a guinea pig retinal slice in low mesopic conditions (G). B. Current voltage relations show smaller calcium currents in the presence of muscimol. C. Activation curves of the cell in (A) reveals V_½_ shifts from −36 to −29 mV with muscimol application (cf. Fig 4F–4I). D–F. Same experiment in A–C in the presence of CNQX (50 μM). Under these conditions, muscimol shifts V_½_ from −21 to −34 mV. G. Graphic to show that the recordings are made from cones. H. A summary plot of ΔV_½_ elicited by muscimol application in control (*n* = 6) and in CNQX (*n* = 4) demonstrates that the polarity of muscimol’s effect is dependent on glutamatergic depolarization of horizontal cells. Underlying data of cells in this figure can be found in S1 Data. Ca_V_ channel, voltage-gated Ca^2+^ channel; CNQX, 6-cyano-7-nitroquinoxaline-2,3-dione; V_½_, half-maximal activation voltage.

In our recordings of the modulation of Ca_V_ channels in cones from retinas maintained in dark mesopic conditions (Figs 1–7), horizontal cells were in a relatively depolarized state. Horizontal cells are depolarized by glutamate, which is released in a graded manner by photoreceptors maximally in darkness, resting under this condition at membrane potentials as high as −30 mV. It is broadly appreciated that reducing glutamatergic transmission with intense illumination or with glutamate receptor antagonists, such as CNQX, horizontal cells hyperpolarize to levels near −60 mV [11]. At the depolarized end of this range of potentials, horizontal cells inhibit photoreceptor Ca_V_ channels, while at the hyperpolarized end they produce disinhibition of those channels [29].

We investigated the effect of muscimol on guinea pig cones with and without CNQX pretreatment under mesopic conditions. Fig 7A–7C shows that muscimol typically causes a 5.6 ± 0.5 mV rightward shift in Ca_V_ channel activation midpoint for these cells (CI: 6.4–4.7; *P* < 0.001; *n* = 7). In retinas pretreated with CNQX, which by itself shifts cone Ca_V_ channel activation midpoint leftward by 5.9 ± 0.8 mV (CI: 4.7–7.2; *P* < 0.001; *n* = 4; S4 Fig), the effect of the GABA agonist changes dramatically. In the presence of CNQX, muscimol application elicited an 11.1 ± 1.5 mV leftward shift in the activation midpoint (CI: 8.6–13.7; *P* < 0.001; *n* = 4; comparison to control: *P* < 0.001; Fig 7D–7F). These findings confirm previous reports that the cone Ca_V_ channel activation curve shifts laterally, depending on horizontal cell membrane potential [10,11,77], and that GABAR-mediated cone inhibition can reverse depending on the membrane potential of horizontal cells [2].

Since NHE cannot account for alkalization of the synaptic cleft, as it does not mediate H^+^ influx [78], it is likely that the disinhibition seen in CNQX is due to horizontal cell GABAR-mediated HCO_3_^−^ efflux, which increases with horizontal cell hyperpolarization. Together with our findings that GABA loses its inhibitory effects on cone Ca_V_ channels in the presence of the NKCC1-blocker bumetanide and the NHE-blockers amiloride and cariporide, this result suggests that the inhibitory effects of GABARs during horizontal cell depolarization balance against the disinhibitory actions during hyperpolarization.

## Discussion

**Fig 8:**
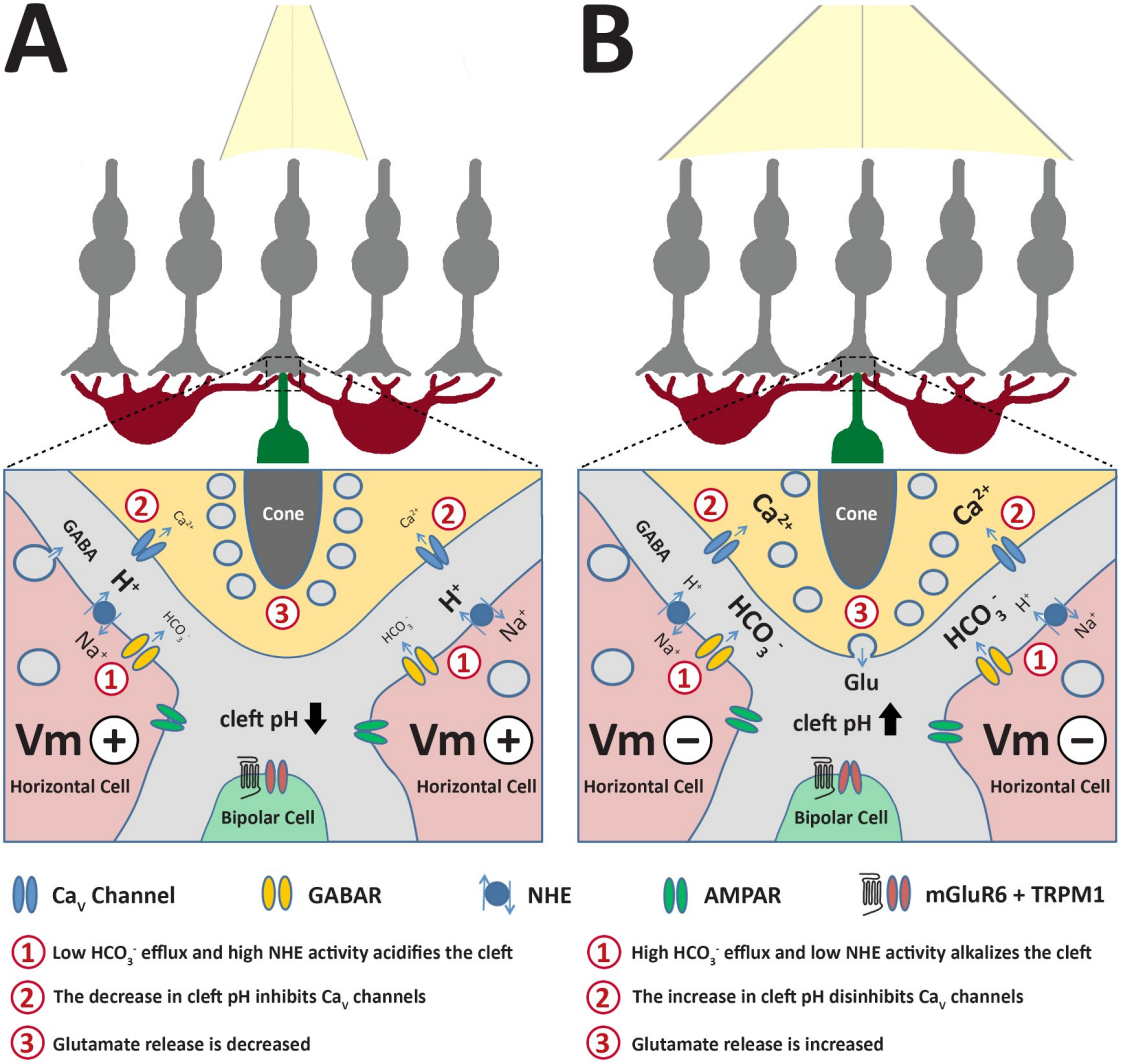
Modulation of cone presynaptic Ca_V_ channels by horizontal cell regulation of cleft pH. A. Acidification of the cleft during horizontal cell depolarization (“Vm+”) in relative dark. HCO_3_^−^ efflux via GABARs decreases due to reduced driving force (E_HCO3_^−^ close to Vm+). Continuous H^+^ extrusion occurs to offset intracellular acidification caused by metabolic activity. Inhibited Ca^2+^ influx into cone reduces glutamate release. B. Alkalization of the synaptic cleft occurs during strong hyperpolarization (“Vm‒”) of the horizontal cells due to increased driving force on HCO_3_^−^ efflux via GABARs and reduced H^+^ efflux, disinhibiting cone Ca_V_ channels and increasing glutamate release. Ca_V_ channel, voltage-gated Ca^2+^ channel; GABAR, GABA receptor; Vm, membrane potential.

These studies establish a novel mechanism of synaptic feedback onto mammalian photoreceptors. A foundation of this feedback mechanism is already well established in nonmammalian and mammalian vertebrates, namely, that horizontal cell depolarization inhibits photoreceptor voltage–gated Ca_V_ channels due to an acidifying pH shift within the synaptic cleft [10,11,13,24]. Our results now reveal the role for GABA in this pH shift in mammalian retina. Specifically, we find that horizontal cells tonically release GABA that activates C1^−^ and HCO_3_^−^ permeable GABAR autoreceptors. Evidence suggests that the effect of this feedback depends on the membrane potential of the horizontal cell. The GABAR-mediated HCO_3_^−^ efflux intrinsically alkalinizes the cleft when the horizontal cell is hyperpolarized, or it adds to depolarization of the cell sufficiently to result in cleft acidification via an overwhelming NHE H^+^ efflux (Fig 8). Our model may account for earlier observations of feedback to photoreceptors in mammalian retina that were previously interpreted as ruling out a role for GABA in direct feedback to cones [14] (see S5 Fig).

### Horizontal cells release GABA

Inconsistent observations regarding the presence of GABA in and its release by horizontal cells have historically obscured its role at this synapse. Many of these discrepancies are due to differences in GABA-uptake and -release mechanisms in mammalian versus nonmammalian horizontal cells [35,79–81]. In nonmammalian vertebrates, horizontal cells take up [82] and release GABA through reversed GABA uptake [80], but mammalian horizontal cells lack a GABA-uptake mechanism [83,84]. In addition, immunostaining for GABA in horizontal cells has proven inconsistent in some mammalian species, possibly a result of masking of epitopes by over-fixation and/or intrinsically low GABA synthesis rates [48].

Notwithstanding the questions concerning the immunohistochemical labeling for GABA in horizontal cells, many lines of evidence support a role for GABA at this synapse in the mammalian retina. Many of the proteins typically associated with vesicular GABA release have been noted in horizontal cells, including L-glutamate decarboxylase and GABA itself [35,48,85,86] as well as VGAT, V-ATPase, multiple SNARE and vesicle proteins, and Ca_V_ channels localized to horizontal cell synaptic processes [2,34,87–89]. Furthermore, vesicle membrane fusion and recycling in horizontal cells is depolarization- and Ca^2+^-dependent [81], and the deletion of VGAT from horizontal cells abolishes horizontal cell inhibitory feedback to photoreceptor Ca_V_ channels [61]. These findings support the contention that mammalian horizontal cells use a vesicular mechanism to release GABA. Our demonstration of horizontal cell autoreception also implies the tonic availability of GABA in the synaptic cleft (Fig 3), even when horizontal cells are chronically hyperpolarized by CNQX, consistent with the lack of an uptake mechanism in horizontal cells.

### GABARs are present on horizontal cell dendritic tips but not cone pedicles

Our immunohistochemical data place ρ-subunit–containing GABARs on the tips of horizontal cell dendrites and axons that make contacts with cone and rod terminals, respectively (Fig 2), and our recordings from the Cx57-VGAT-KO mouse suggest that these GABARs are autoreceptors (Fig 3). GABARs containing ρ-subunits have high affinity for GABA and are nondesensitizing, making them well suited for generating tonic GABA currents [90]. These findings fit earlier reports of a GABA-activated conductance in mammalian horizontal cells [2,40]. While our observations show that horizontal cell GABA autoreceptors, rather than cone GABARs, mediate horizontal cell-to-cone feedback, some studies suggest mammalian cones may express GABAR subunits [91–93]. While direct horizontal cell-to-photoreceptor GABA transmission is reported in the retinas of several nonmammalian vertebrates [16,18,20], we saw neither a conductance increase nor decrease in cones in the voltage range of −80 to −50 mV, which is outside the range of Ca_V_ channel activation and inconsistent with direct activation of cone GABARs with GABA. Reports that the expression of GABARs changes throughout the circadian cycle may indicate that different feedback mechanisms operate, more or less robustly, at different times of the light cycle [94].

Our investigations have not yet tested the pH sensitivity of other ionic conductances present at the cleft, such as those in photoreceptors, in which Ca^2+^-activated chloride channels are known to be in proximity to the cleft [95] (but not HCN1 channels [96] or Kx channels [97]), and those in horizontal cells in which we expect that some of the Ca_V_ channels face the cleft. While we do not yet know of any pH sensitivity of the ρ2-subunit–containing GABARs shown here to be situated at the cleft, it is important to note that rat and human ρ1 GABAR currents are pH dependent, with current amplitudes dropping 30% as pH is reduced from 7.8 to 7.0, an effect that becomes greater at low GABA concentrations [98]. It will be crucial to account for the pH-induced changes of this GABAR and all other relevant channels and transporters to fully understand how cleft pH modulates this form of signaling.

### Depolarized neurons extrude protons

Depolarized cells produce and extrude acid due to the metabolic activity required to maintain concentration gradients for Na^+^, K^+^, Ca^2+^, and other ions. The active transport of these ions against their gradients, requiring ATP for Na^+^/K^+^-ATPase and plasma membrane Ca^2+^-ATPase (PMCA), is acknowledged to be the largest energy expenditure for neurons [99,100]. At excitatory synapses in which Na^+^ influx and K^+^ efflux can be protracted, and especially so in the present case in which horizontal cells are in a tonically depolarized state during low illumination due to the continuous release of glutamate from photoreceptors, the energy cost of active ion transport to maintain transmembrane ion gradients is high [32]. The energy requirements of the retina are higher in the dark than in the light, and the retina relies on glycolysis and oxidative phosphorylation to supply ATP [99]. It has long been appreciated that these high metabolic costs and their dependence on illumination contributes to the sustained, low bulk pH in the outer retina in the dark and its increase during illumination [101–103].

Evidence for the extrusion of protons into the extracellular space, occurring significantly via NHEs and the resultant extracellular acidification, was seen when we blocked NHEs and inhibitory feedback was lost and there was no further regulation by TPMPA (Fig 6). This finding that horizontal cell GABAR-mediated cleft acidification is dependent on NHE proton extrusion supports the role of depolarization mediated production and extrusion protons in horizontal cell feedback; however, our experiments did not resolve any temporal features of this process. We consider that this voltage–metabolic relation contributes continuously to the background acidification that inhibits photoreceptor Ca_V_ channels [32] and encompasses the contribution of horizontal cell regulation of cleft pH as a function of their membrane potential. Depolarization induces further cleft acidification, and cleft pH can rapidly alkalinize during hyperpolarization due to voltage driven efflux of HCO_3_^−^ via GABARs.

### Horizontal cell voltage drives cleft pH changes via counterbalanced mechanisms

Our findings demonstrate that GABA modulates cone Ca_V_ channel activation by altering cleft pH (Fig 1). Muscimol application to horizontal cells under mesopic conditions, as well as activation of exogenous ligand-gated Cl^−^/HCO_3_^−^ channels (PSAM-GlyRs), resulted in shifts of Ca_V_ channel activation curve midpoints to more positive potentials. We link this inhibitory effect of horizontal cell GABARs to their depolarization of horizontal cells (Fig 5) and the ensuing activation of proton-extruding NHEs (Fig 6).

While GABAR-mediated Cl^−^/HCO_3_^−^ efflux can depolarize horizontal cells and increase NHE activity, the HCO_3_^−^ efflux may also provide an alkalinizing influence in the synaptic cleft. Due to NHE H^+^ efflux, constitutive depolarization-induced acidification is strong enough to overpower HCO_3_^−^-induced alkalization under scotopic and mesopic conditions. Therefore, when GABARs are blocked with picrotoxin or TPMPA and the depolarizing contribution provided by these conductances is lost, the net effect is an increase in pH due to hyperpolarization, inhibition of NHE H^+^ efflux, and increase in HCO_3_^−^ efflux. In bright light, however, when horizontal cell membrane potential is very negative, even with a tonic presence of GABA, the strong driving force on HCO_3_^−^ efflux increases and helps alkalinize the cleft (Fig 7).

The dual effects of GABA, depolarizing and alkalinizing, work in concert to extend the functional voltage range of horizontal cells. The linear voltage-dependent flux of HCO_3_^−^ afforded by GABARs extends the range of pH changes to more hyperpolarized values of horizontal cell membrane potential. The Cl^−^ conductance amplifies depolarizations into the range of steeply voltage-dependent acidification provided by NHEs at positive voltages.

HCO_3_^−^ transport would be expected to play a role in the relatively positive equilibrium potential for HCO_3_^−^ in horizontal cells, mediated by numerous anion transporters that commonly take part in the regulation of intracellular pH as well as proton transporters, as the changes they make to intracellular pH also change [HCO_3_^−^] levels [104]. 4,4'-Diisothiocyano-2,2'-stilbenedisulfonic acid (DIDS), a broad spectrum anion transport blocker, has been recently shown to play a key role in feedback in salamander retina [52]. The activation of a large anion conductance in a cell is expected to alter regulation of HCO_3_^−^ and Cl^−^ levels, as shown earlier as a rapid fall in intracellular pH during stimulation with GABA [41]. HCO_3_^−^ transport mechanisms play important roles in both intracellular and extracellular pH regulation [105–107]. and investigation of their function in feedback is warranted. For example, AE3 (Slc4a3), a Cl^−^/ HCO_3_^−^ exchanger, is expressed extensively in horizontal cells and Müller cells [108], and Slc4a3 null mice have no b-wave, possibly reflecting an effect on horizontal cell feedback [109].

While our investigations implicated H^+^ and HCO_3_^−^ fluxes in generating pH changes, further identification and separation of these components would add to the understanding of their specific functional properties in horizontal cell signaling. Block of extracellular carbonic anhydrase (eCA) enhances pH changes caused by H^+^ fluxes and suppresses pH changes caused by HCO_3_^−^ fluxes [41,110,111]. Not only is eCA expressed in the retina [30,112,113], but the effects of its inhibition on feedback to photoreceptors have been examined in fish and salamanders [30,52]. Block of eCA suppressed the normal cone presynaptic Ca_V_ channel disinhibition during horizontal cell hyperpolarization, whereas presynaptic inhibition during horizontal cell depolarization was unchanged [30]. These results favor a HCO_3_^−^-mediated mechanism [41,110,111].

### Dual mechanisms account for surround inhibition of cones

Baylor and colleagues first recorded feedback inhibition in turtle cones, finding that surround illumination produced a delayed depolarization that opposed the hyperpolarizing, direct response to light [114]. Since these early measurements, horizontal cells have been thought to provide a critical source of lateral inhibition that contributes to center-surround receptive field formation. Here, we describe how this depolarization of cones is instantiated by the mechanisms of a pH-mediated feedback in which horizontal cell hyperpolarization, produced by surround illumination, results in alkalization of the synaptic cleft and an increase in Ca^2+^ conductance. The best perspective of the function of horizontal cell feedback to cones in mammals is provided in the voltage clamp recordings of the current induced by “pure” surround illumination upon a standing spot of light [14] (see S5 Fig). In that report, voltage clamping the membrane potential of the central cone at −40 mV showed that surround illumination produced an inward current identified to arise from increased Ca_V_ channel activation with subsequent contribution from Ca^2+^-activated Cl^−^ channels [14]. These surround evoked inward currents persisted in picrotoxin and GABA, albeit with altered magnitudes. Interpreted within the context that the GABARs would be on cones, not horizontal cells, Verweij and colleagues’ (2003) results appeared to rule out a role for GABA. That is, their findings were not explainable by a direct action of GABARs at the cone (which would have led to block of surround-induced inward current changes in both picrotoxin and GABA). In contrast, their results appear to fall within the GABAergic autaptic feedback mechanism described in this report. We consider that the recordings in macaque show the full extent of the actions of feedback from horizontal cells to voltage-clamped cones [14] and recognize the great differences of these actions from those seen in nonmammalian vertebrates, in which cones do express GABARs [16,20].

Recent advances that reveal the functional role of horizontal cells in retinal visual processing have been made by recording ganglion cell responses to light following ablation, silencing, and shunting of horizontal cells. In one study, ablation of horizontal cells reduced surround inhibition, ON and OFF subtype diversity and adaptation, and altered spatial frequency tuning [4]. In another, genetic deletion of glutamate receptor expression in horizontal cells, which makes them incapable of responding to photoreceptor glutamate signals, similarly altered the surround-receptive field structure in α-OFF transient ganglion cells and suggested a role of horizontal cells in adjusting ganglion cell dynamic range [6]. And in another, activation of PSAM-GlyRs in horizontal cells was also shown to alter ganglion cell light responses [7]; however, this third approach is markedly different, as it should result in horizontal cell depolarization (cf. Fig 4). Unless the anionic conductance is so large as to fully shunt the horizontal cell voltage to a value close to E_Cl_ and keep it there, the altered responses of ganglion cells, which included suppression of transient excitatory inputs, enhancement of sustained ON-type responses, suppression of all OFF-type responses, and no effect on fast ganglion cell responses, arise from a different alteration of horizontal cell activity than that caused by silencing with hyperpolarization.

The advantages of this form of pH-mediated feedback over more direct means of transmitter-mediated inhibition are unclear. Synaptic gain control to maintain temporal response fidelity over a broad range of light levels should require feedback having fast kinetics, which the tonic HCO_3_^−^ permeability offers. Feedback mediated by pH changes would provide filtering matching the slow membrane potential changes recorded in horizontal cells in response to broad changes in contrast over extended areas and time, with additional slow components mediated by changes to buffering strength and GABA concentration. Additional modes of horizontal cell feedback, such as that offered by the ephaptic effect [115], ATP release [25], and very localized positive feedback [116] may provide a range of kinetic properties to optimize contrast sensitivity over smaller temporal and spatial scales.

As with GABA- and pH-mediated signaling in horizontal cell feedback, changes in extracellular acidity in response to GABAergic activity have been noted throughout the brain [43–45]. Comparable mechanisms affect synaptic acidification at GABA and glycinergic synapses elsewhere in the CNS. This pH-mediated action of GABA in the horizontal cell-to-photoreceptor synapse represents a novel means of inhibitory signaling in a graded potential network, although such a mechanism is not unique to the retina [46].

## Materials and methods

Electrophysiological experiments were performed in accordance with the guidelines for the welfare of experimental animals issued by the United States Public Health Service Policy on Human Care and Use of Laboratory Animals (2002) and the University of California at Los Angeles (UCLA) Animal Research Committee.

### Patch clamp recording from cone photoreceptors and horizontal cells in retinal slices

Ca_V_ channel currents were measured in cones from mouse, rat, and guinea pig retinal slices and in mouse horizontal cells in slices using standard whole-cell patch clamp techniques under IR illumination. The Cx57-iCre^+/−^:: R26tdTomato^+/−^ (Cx57-tdTomato) transgenic mouse line [37] was used for recording horizontal cells in slices since only horizontal cells express tdTomato fluorescence, allowing positive identification of the cells for patch clamping. Adult C57BL/6J mice and Sprague Dawley rats (Charles River Lab, Wilmington, MA) were deeply anesthetized with 1%–3% isoflurane (IsoFlo, Abbott Laboratories, Abbott Park, IL) and decapitated. For euthanizing guinea pigs, IP injection of pentobarbital (Fatal plus, Vortech, Dearborn, MI) produced deep anaesthesia, after which thoracotomy was performed. The eyes were then enucleated in dim light, and the anterior portion of the eye including the lens removed. The resulting eyecup was trimmed, and a section of retina with scleral backing was placed vitreal side down on a piece of filter (Millipore; 2 × 5 mm, type GS, 0.2-μm pores). After the retina had adhered to the filter, the sclera was peeled away and the retina and filter paper were cut into 150–200 μm slices using a tissue chopper (Stoelting Tissue Slicer, Stoelting Co., Wood Dale, IL) mounted with a razor blade (No. 121–6; Ted Pella Inc., Redding, CA) and the slices rotated 90° to facilitate viewing of the retinal layers. Slices were superfused via a gravity driven fast flow system (ALA, Farmingdale, NY) with a solution containing (in mM) 120 NaCl, 2 CaCl_2_, 3 KCl, 1 MgCl_2_, 1.2 NaH_2_PO_4_, 10 glucose, 25 NaHCO_3_, bubbled continuously with 95% O_2_−5% CO_2_. Room-temperature (21–24 °C) solutions were superfused. To reduce movement of the slices, the rate of fluid exchange was adjusted so that the bath volume was changed with a time constant of <15 sec. For Ca_V_ channel current recordings, the internal solution usually contained (mM) 110 CsMeSO_4_, 2.8 NaCl, 4 EGTA, 5 TEACl, 4 Mg-ATP, 0.3 Na_3_-GTP, 20 Hepes, 10 Na_2_-phosphocreatine, pH 7.25.

Horizontal cells of Cx57-tdTomato mice in slices were patch clamped in the same external solution as cones but with pipette solutions containing (mM) 110 CsMeSO_4_, 2.8 NaCl, 4 EGTA, 5 TEACl, 4 Mg-ATP, 0.3 Na_3_-GTP, 20 Hepes, 10 Na_2_-phosphocreatine, pH 7.25. Some recordings were made with a “negative 30 mV E_Cl_” intracellular solution containing 109 K gluconate, 41 KCl, 4 Mg-ATP, 0.3 Na-GTP, 10 Na_2_-phosphocreatine, 4 EGTA, 20 Hepes, at pH 7.25 with NaOH (Fig 5D) or 140 CsCl, 0.1 CaCl_2_, 1 EGTA, 10 Hepes, 3 Mg-ATP, 0.2 Li-GTP, and 8 phosphocreatine, at pH 7.2 with CsOH (S2A Fig). In some recordings, gramicidin-perforated patch techniques were used, with Kgluconate internal solution containing 41 mM chloride. Liquid junction potential (LJP) errors were corrected in all figures after being calculated and measured. For example, for the CsMeSO_4_ intracellular solution, a −8.7 mV LJP was calculated.

Patch electrodes with 5 to 10 MΩ tip resistance were pulled from fire-polished borosilicate glass capillary tubes using a micropipette puller (Sutter Instrument, Novato, CA). The bath reference electrode consisted of an AgCl wire in a side chamber. Cell voltage was clamped with a MultiClamp 700B amplifier (Molecular Devices, Sunnyvale, CA) using whole cell capacitance and series resistance compensation. Current signals were filtered at 5 kHz and digitized at 10 kHz with a Digidata 1440A for storage on the hard disk of a computer running pCLAMP 9 acquisition software (Molecular Devices, Sunnyvale, CA).

### Patch clamp recording from isolated horizontal cells

Currents were measured in isolated Cx57-tdTomato mouse horizontal cells using whole-cell patch clamp. Mice were deeply anesthetized with 1%–3% isoflurane, decapitated, the eyes were enucleated, and the anterior portion of an eye including the lens removed. The retina was removed and incubated in Hanks’ Balanced Salt Solution (HBSS−/−; SH30031.03; Hyclone, Logan, UT) containing 18 U/ml papain and 100 U/ml DNase I (Worthington Biochemical, Freehold, NJ) at 37 °C for 40 min. Isolated cells were obtained by gentle trituration after digestion. The cells were kept in Dulbecco’s Modified Eagle Medium (DMEM; Life Technologies, Grand Island, NY) with 10% Fetal Bovine Serum (FBS; Life Technologies) and penicillin/streptomycin (1X, Invitrogen) in a 5% CO_2_ incubator at 37 °C. For identification of the fluorescent horizontal cells and initial patch clamp recording, the solution was changed to a standard bathing solution containing (in mM) 138 NaCl, 3 KCl, 2 CaCl_2_, 1.25 NaH_2_PO_4_, 1 MgCl_2_, 10 glucose, and 10 Hepes, adjusted to pH 7.4 with NaOH, which was delivered via a gravity-driven fast flow system. In some recordings, gramicidin-perforated patch techniques were used, with Kgluconate internal solution containing 41 mM chloride. Room-temperature (21–24 °C) solutions were superfused via a gravity-driven system. Recording techniques used for the isolated cells were essentially identical to those described above for neurons in slices, with the exception that different intracellular solutions were used in some recordings.

### Statistics

All data are reported as the mean ± standard error of the mean (SEM). Graphing and statistical analyses were performed using R (R Foundation for Statistical Computing). Due to the limited sample sizes of some experiments, bootstrapping methods were used to generate confidence intervals and *P* values [117]. This approach was chosen over permutation tests (and related ranking-based tests such as Wilcoxon signed-rank) because the effects in this paper were seen in every cell and under such circumstances, permutation tests only report number of samples. Confidence intervals (CI) were determined using random sampling with replacement (100,000 replicates; percentile method) [118]. To estimate a null distribution for P value calculation, the mean of the resampled population was shifted to zero. For unpaired conditions, in each replicate we calculated the difference between the mean of each resampled group; for paired conditions, in each replicate, we summed the resampled differences. *P* values are noted as *P* < 0.05, *P* < 0.01, *P* < 0.001, or actual value when above 0.05. *P* values of less than 0.05 were considered statistically significant and are denoted in the figures by an asterisk.

### Immunohistochemical labeling

Following deep anaesthesia as described in previous sections, animals of hemizygous mouse lines *Cx57-iCre*^−*/*−^:: *VGAT*^*flox/flox*^ (WT), *Cx57- iCre*^*+/*−^:: *VGAT*^*flox/flox*^ (VGAT KO), *Cx57-iCre*^*+/*−^:: *R26tdTomato*^*+/*−^ (Cx57-tdTomato), and *Cx57- iCre*^*+/*−^ (Cre) were decapitated, the eyes enucleated, and the anterior chamber and lens removed. The eyecups were immersion-fixed in 4% (w/v) paraformaldehyde (PFA) or 2% PFA, 75 mM L-lysine, and 10 mM Na-periodate (2% PLP) in 0.1 M phosphate buffer (PB), pH 7.4, for 15–30 min, cryoprotected in 30% sucrose, and sectioned vertically at 12–14 μm on a cryostat onto gelatin-coated slides. Immunostaining was performed using the indirect fluorescence method [36]. In brief, retinal sections were incubated in a blocking solution containing 10% normal goat serum (NGS), 1% bovine serum albumin (BSA), 0.5% Triton X-100, 0.05% sodium azide (NaN_3_) in 0.1M PB, pH 7.4 for 1 hour. The primary antibody was diluted in 3% NGS, 1% BSA, 0.5% Triton X-100, 0.05% NaN_3_, in 0.1 M PB, for 12 to 16 hours at room temperature. The specific immunolabeling was visualized using Alexa Fluor 488-, 568- or 594-conjugated anti-rabbit or mouse secondary antibodies (Invitrogen, Grand Island, NY) at 1:500–1:1,000 dilutions for 120 min at room temperature. The immunostaining was examined on a Zeiss 880 Laser Scanning Microscope employing Airyscan (Carl Zeiss, Inc., Thornwood, NY) with Zeiss C-Apochromat 40x (1.2 NA) or C-Apochromat 63x (1.2 NA) corrected water or Plan-Apochromat 63x (1.4 NA) oil objectives. Confocal images were analyzed using the Zeiss LSM 710/880 proprietary software (Zen version 2.3). Intensity levels and brightness/contrast were adjusted in Adobe Photoshop CS6 v.12.2 (Adobe Systems, San Jose, CA). Antibodies used included rabbit anti-GABA (A) ρ2 receptor polyclonal antibodies (Alomone AGA-007, 1:1,000, Israel) and anti-VGAT (Synaptic Systems #131 011, 1:1,000, Germany). The specificity of immunolabeling by the GABAR ρ2 subunit antibody was confirmed by preadsorption with the antigenic peptide, a condition under which labeling was found to be absent (S6 Fig). Mouse monoclonal antibodies against calbindin (Sigma C9848, 1:2,000) were used to label horizontal cells [36,61,119].

### Expression of PSAM-GlyR in horizontal cells

We induced expression of PSAM-GlyR in Cx57-iCre^+/−^ mouse horizontal cells by using a Cre-dependent construct in AAV-7m8 that penetrates deeply into the retina following an intravitreal injection [120] for cell modulation via activation of PSAM-GlyR [63]. The effector molecule, PSEM^308^ (Apex Scientific, Stony Brook, NY) activates the PSAM-GlyR at 0.2 μM. This ligand does not bind to known naturally occurring receptors at this concentration [64] nor did it have any effect on control horizontal cells. We used a EF1α promoter in the AAV-7m8 construct (AAV-7m8-[EF1α]-FLEX-PSAM/GlyR-IRES-GFP) (Fig 5A). Following a 6–10-week survival post intraocular injections of anesthetized mice, horizontal cells and their processes robustly express GFP, the reporter for the AAV-7m8 construct. Horizontal cell identity was confirmed by calbindin immunoreactivity, a marker of horizontal cells [47]. Horizontal cell infection based on colocalization of GFP and calbindin immunoreactivity was greater than 90% (*N* = 3 retinas) after a 42-day survival. Horizontal cells expressing AAV-7m8-PSAM-GlyR, recorded with a CsCl-filled patch electrode to give an artificial Cl¯ reversal potential near 0 mV, show a conductance increase reversing at +3 mV during application of PSEM (S2A Fig). Light response waveforms in another horizontal cell (S2B Fig), recorded with a low Cl^−^ intracellular solution giving a Cl equilibrium potential near −67 mV, show hyperpolarization of the dark potential and reduction of the light induced hyperpolarization (tan trace) during PSEM application, compared to the control trace recorded prior to applying this ligand (blue trace).

### Drugs and chemicals

All chemicals and reagents, unless otherwise noted, were obtained from Sigma-Aldrich (St. Louis, MO). TPMPA, PTX, bumetanide, muscimol, gabazine, strychnine, amiloride, and CNQX were obtained from Tocris (Ellisville, MO). Drugs and reagents were prepared in double-distilled water either as stock solutions (frozen at −20 °C) or prepared fresh. Superfused drugs normally produced their full effects in approximately 1 min, but in cases in which no response was seen, a limit of 5 min was deemed sufficient to conclude an absence of action.

## Supporting information

S1 Fig GABAR ρ2 subunits remain expressed and localized to horizontal cell endings in the Cx57-VGAT-KO mouse. (A) Calbindin immunolabeling in the OPL identifies horizontal cells, including cell bodies (*), processes (dendrites and axons), and endings. (B) GABAR ρ2 subunit immunoreactivity in the OPL. (C) Merge image depicts colocalization of GABAR ρ2 subunits (blue) with calbindin (red) immunolabeling. Small arrow points to horizontal cell dendritic endings that contact cone pedicles. Large arrows point to horizontal cell axon terminals that contact rod spherules. Single optical section, Airyscan processed. Scale bar = 10 μm. GABAR; GABA receptor; KO, knockout; OPL, outer plexiform layer; VGAT, vesicular GABA transporter.
(TIF)

S2 Fig Chemogenetic modulation of mouse horizontal cells. (A) Conductance increase with PSEM^308^ (10 μM) application to PSAM-GlyR–expressing isolated, transduced horizontal cells identified via the viral construct’s GFP reporter. Voltage ramp I–V relations recorded before and during PSEM superfusion, showing increased conductance at all voltages, reversing just positive 0 mV. E_Cl_ = 0 mV. (B) Responses to light-response waveform stimulation (during bar) in another horizontal cell show hyperpolarization of the dark potential and reduction of the induced hyperpolarization (tan trace) during 10 μM PSEM application compared to the control trace recorded prior to applying this ligand (blue trace). Note that E_Cl_ was set to −60 mV in this recording, not the value of −30 mV recorded with gramicidin-perforated patch clamp in Fig 5. Light-response waveform stimulation (3 X 10^13^ photons/s/μm^2^) [77] was used to isolate the effect of the PSEM conductance increase from confounding actions of the inhibitory feedback loop. Additional cells showing similar responses were observed but not analyzed. E_Cl_, chloride equilibrium potential; GFP, green fluorescent protein; GlyR, glycine receptor; I–V, current–voltage; PSAM, pharmacologically selective actuator module; PSEM, pharmacologically selective effector molecule.
(TIF)

S3 Fig Blocking Na^+^/H^+^ exchangers with amiloride disinhibits cone Ca_V_ channels and eliminates the disinhibitory effect of TPMPA. A. Patch clamp recording of a mouse cone. B. Currents elicited by the voltage steps shown in the absence (top) and presence (bottom) of the NHE-blocker amiloride (30 μM). C. I–V relations show larger Ca_V_ channel currents, activating at more negative voltages, in the presence of amiloride. D. Shift of the activation curve of the cell in (B) to more negative potential during amiloride application. E–H. Same paradigm as the experiment in A–D but retinal slice pretreated (30 min) and bathed continuously with 10 μM amiloride. Under these conditions, TPMPA fails to shift Ca_V_ channel activation curve to more negative potentials (H). Underlying data of cells in this figure can be found in S1 Data. Ca_V_ channel, voltage-gated Ca^2+^ channel; I–V, current–voltage; NHE, Na+/H+ exchanger; TPMPA, (1,2,5,6-tetrahydropyridin-4-yl)methylphosphinic acid.
(TIFF)

S4 Fig The glutamate receptor antagonist CNQX disinhibits guinea pig cone Ca_V_ channels. A. Currents elicited by voltage steps shown in a cone before (top) and during (middle) 50 μM CNQX application and in both 100 μM muscimol and 50 μmM CNQX (bottom). B. I–V relations show larger Ca_V_ channel currents in the presence of CNQX. C. The cone Ca_V_ channel activation curve shifts to a more negative potential during CNQX application (−20.3 mV to −24.5 mV). D. I–V relations show even larger Ca_V_ channel currents in the presence of CNQX and muscimol. E. The cone Ca_V_ channel activation curve shifts to a more negative potential during muscimol application in a cone bathed in CNQX (−24.5 mV to −31.9 mV). Underlying data of cells in this figure can be found in S1 Data. Ca_V_ channel, voltage-gated Ca^2+^ channel; CNQX, 6-cyano-7-nitroquinoxaline-2,3-dione; I–V, current–voltage.
(TIF)

S5 Fig Modulation of surround light-response current amplitude by picrotoxin and GABA in macaque cones. (Redrawn from Figure 4 of Verweij and colleagues [2003] [14]). Macaque cones, voltage clamped near −40 mV, respond with an inward current when full field illumination (“full;” 0.5 s) was added to continuous spot illumination (“spot”). The control current increase (“cont”) was attributed to an increase in Ca_V_ channel and Cl(Ca) currents. In this figure, superfusion with picrotoxin (200 μM) made the inward current larger (left, “picro”) and GABA (500 μM) made the current response smaller (right), similar to the relative Ca_V_ channel current amplitude changes recorded under voltage clamp at −40 mV in cones from mouse and guinea pig during picrotoxin and muscimol superfusion in the present report. The responses to picrotoxin and GABA in macaque cones are not easily explained as being due to the presence of GABARs on cones but are what would be predicted were GABARs on horizontal cells producing changes in cleft pH to alter Ca_V_ channel activation as described in the present report. Ca_V_ channel, voltage-gated Ca^2+^ channel; Cl(Ca), Ca^2+^-activated chloride channel; GABAR, GABA receptor.
(TIF)

S6 Fig Specific immunolabeling by the GABAR ρ2 subunit antibody is completely blocked by preadsorption with the antigenic peptide. (A) GABAR ρ2 immunolabeling in the OPL of mouse retina with 1:1,000 dilution of the ρ2 antibody (AGA-007, Alomone, Jerusalem, Israel). (B) Immunolabeling by GABAR ρ2 antibody (1:1,000) preincubated with the 10^−5^ to 10^−7^ M (shown 10^−6^ M) antigenic peptide ([C]RKRWTGHLETSKPSH, amino acid residues 51–65 of rat GABAR ρ2, accession P47742) for 3.5 hours at 4 °C showed no specific immunoreactivity. Images obtained on a Zeiss LSM 880 with a Plan-Apochromat 63x / 1.4 Oil objective. Projection of 11 optical sections of 0.4 μm, z-step = 0.10 μm, Airyscan processed. Scale bar = 10 μm. GABAR, GABA receptor; LSM, laser scanning microscope; OPL, outer plexiform layer.
(TIF)

S1 Table Species guide to patch clamp data figures. The species stated were used in electrophysiological experiments in cell types listed for the listed figure. In all immunohistochemical investigations, mouse retina was used.
(DOCX)

S2 Table Change of cone conductance (ΔG) in response to GABAergic drugs. Picrotoxin did not cause a change in the resting cone conductance measured between −90 and −60 mV in rats, mice, or guinea pigs. The same result (no change in conductance) was obtained in rats when Hepes was present in the bath. Muscimol, TPMPA, gabazine, and strychnine tested on guinea pig did not change cone conductance. TPMPA did not produce a change in the resting cone conductance measured mice. The slope conductance of lines fit to the I–V relation of each cone between −90 and −60 mV were compared with F-tests and adjusted R2. I–V, current–voltage; TPMPA, (1,2,5,6-tetrahydropyridin-4-yl)methylphosphinic acid.
(DOCX)

S1 Data Supplementary excel files for all cell analyses. Data for each cell represented in Figs 1F, 1G, 1I, 1J, 2B, 2C, 2E, 2F, 2H, 3I, 3J, 3M, 3N, 4G, 4H, 5H, 5I, 5L, 5M, 6C, 6D, 6G, 6H, 7B, 7C, 7E, 7F and 7H; S3B–S3E and S4B–S4E Figs are included in a single excel file as multiple sheets, labeled Figs 1 through 7, S3 and S4 Figs. Data from individual cells underlying statistical analyses described in the text that stand in support of each figure, but which are not shown in the figure, are included on sheets labeled “assoc. with Fig *N*”, in which *N* refers to the associated text figure presented.
(XLSX)

## Abbreviations

AE: anion exchanger
CA: carbonic anhydrase
Ca_V_: channel, voltage-gated Ca^2+^ channel
Cl^−^: chloride
CNQX: 6-cyano-7-nitroquinoxaline-2,3-dione
DIDS: 4,4'-diisothiocyano-2,2'-stilbenedisulfonic acid
eCA: extracellular carbonic anhydrase
E_Cl_^−^: chloride equilibrium potential
E_HCO3_^−^: equilibrium potential for HCO_3_^−^
GABAR: GABA receptor
GAD: glutamic acid decarboxylase
GFP: green fluorescent protein
GlyR: glycine receptor
IRES: internal ribosome entry site
I–V: current–voltage
MCT: monocarboxylic acid transporter
NBC: Na^+^/HCO_3_^−^ cotransporter
NCBE: Na^+^/Cl^−^/HCO_3_^−^ exchanger
NDCBE: Na^+^-driven Cl^−^/HCO_3_^−^ exchanger
NHE: Na^+^/H^+^ exchanger
NKCC: Na^+^/K^+^/Cl^−^ cotransporter
OPL: outer plexiform layer
pH_i_: intracellular pH
PSAM: pharmacologically selective actuator module
PSEM: pharmacologically selective effector molecule
TPMPA: (1,2,5,6-tetrahydropyridin-4-yl)methylphosphinic acid
V_½_: half-maximal activation voltage
V-ATPase: vesicular ATPase
VGAT: vesicular GABA transporter
