# Supplementary figures and images for: Novel hybrid action of GABA mediates inhibitory feedback in the mammalian retina
Source: PLoS Biol. 2019 Apr 1;17(4):e3000200. doi: 10.1371/journal.pbio.3000200 (PMC6459543; doi:10.1371/journal.pbio.3000200)

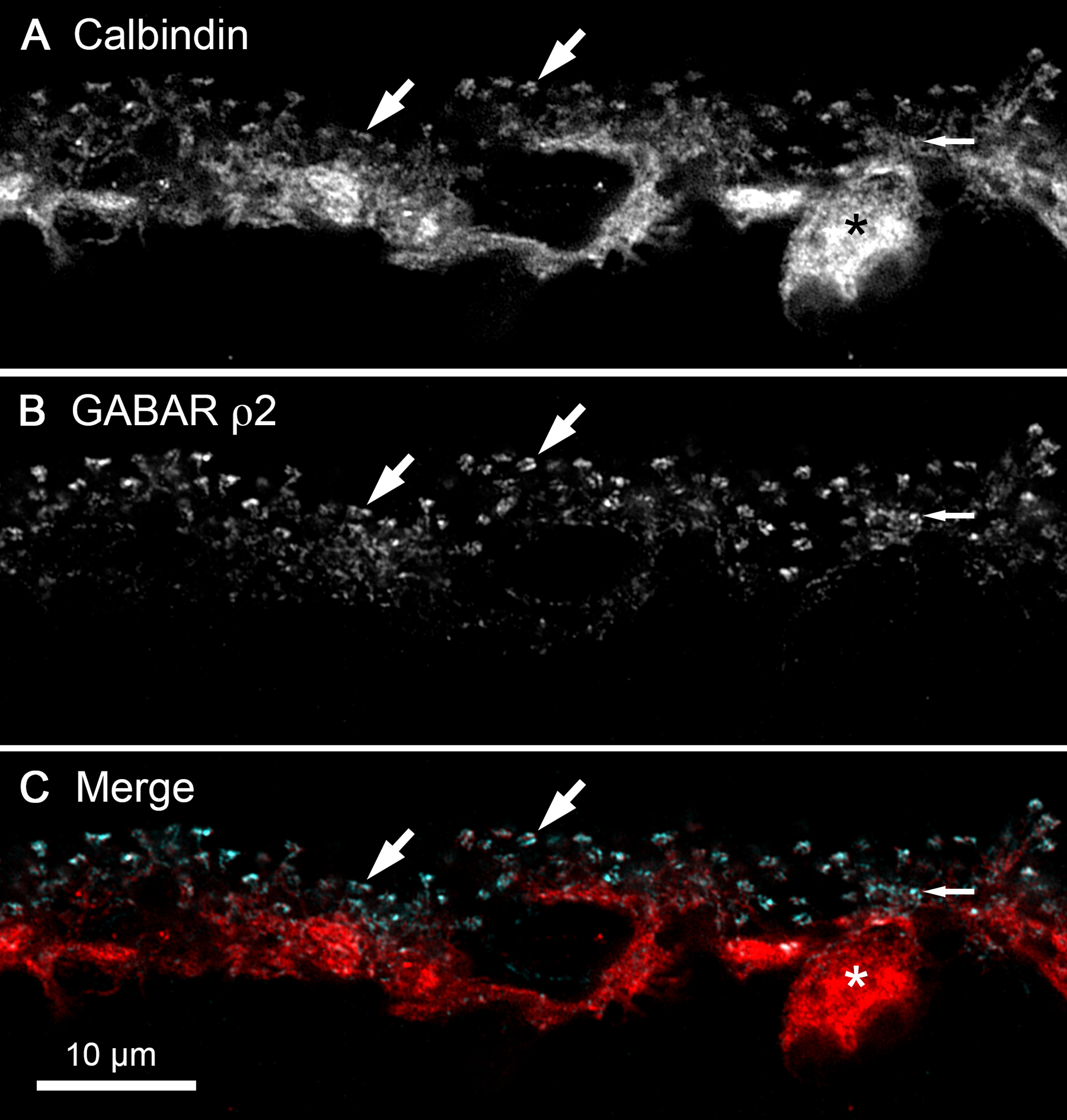

Supplement: S1 Fig — (A) Calbindin immunolabeling in the OPL identifies horizontal cells, including cell bodies (*), processes (dendrites and axons), and endings. (B) GABAR ρ2 subunit immunoreactivity in the OPL. (C) Merge image depicts colocalization of GABAR ρ2 subunits (blue) with calbindin (red) immunolabeling. Small arrow points to horizontal cell dendritic endings that contact cone pedicles. Large arrows point to horizontal cell axon terminals that contact rod spherules. Single optical section, Airyscan processed. Scale bar = 10 μm. GABAR; GABA receptor; KO, knockout; OPL, outer plexiform layer; VGAT, vesicular GABA transporter. (TIF) [file pbio.3000200.s001.tif]

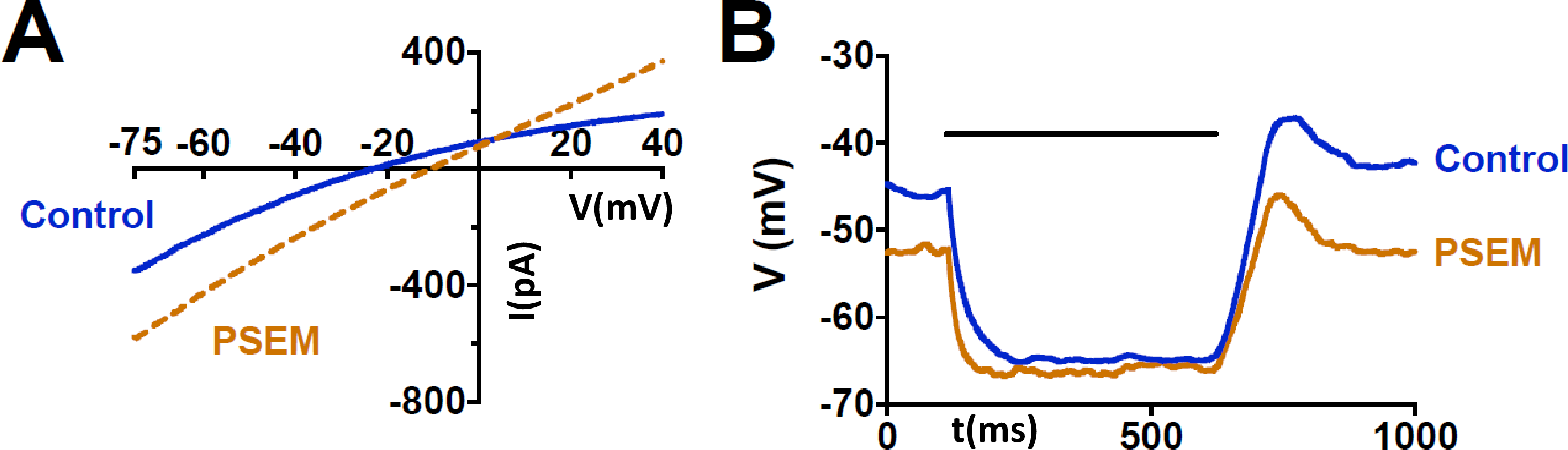

Supplement: S2 Fig — (A) Conductance increase with PSEM308 (10 μM) application to PSAM-GlyR–expressing isolated, transduced horizontal cells identified via the viral construct’s GFP reporter. Voltage ramp I–V relations recorded before and during PSEM superfusion, showing increased conductance at all voltages, reversing just positive 0 mV. ECl = 0 mV. (B) Responses to light-response waveform stimulation (during bar) in another horizontal cell show hyperpolarization of the dark potential and reduction of the induced hyperpolarization (tan trace) during 10 μM PSEM application compared to the control trace recorded prior to applying this ligand (blue trace). Note that ECl was set to −60 mV in this recording, not the value of −30 mV recorded with gramicidin-perforated patch clamp in Fig 5. Light-response waveform stimulation (3 X 1013 photons/s/μm2) [77] was used to isolate the effect of the PSEM conductance increase from confounding actions of the inhibitory feedback loop. Additional cells showing similar responses were observed but not analyzed. ECl, chloride equilibrium potential; GFP, green fluorescent protein; GlyR, glycine receptor; I–V, current–voltage; PSAM, pharmacologically selective actuator module; PSEM, pharmacologically selective effector molecule. (TIF) [file pbio.3000200.s002.tif]

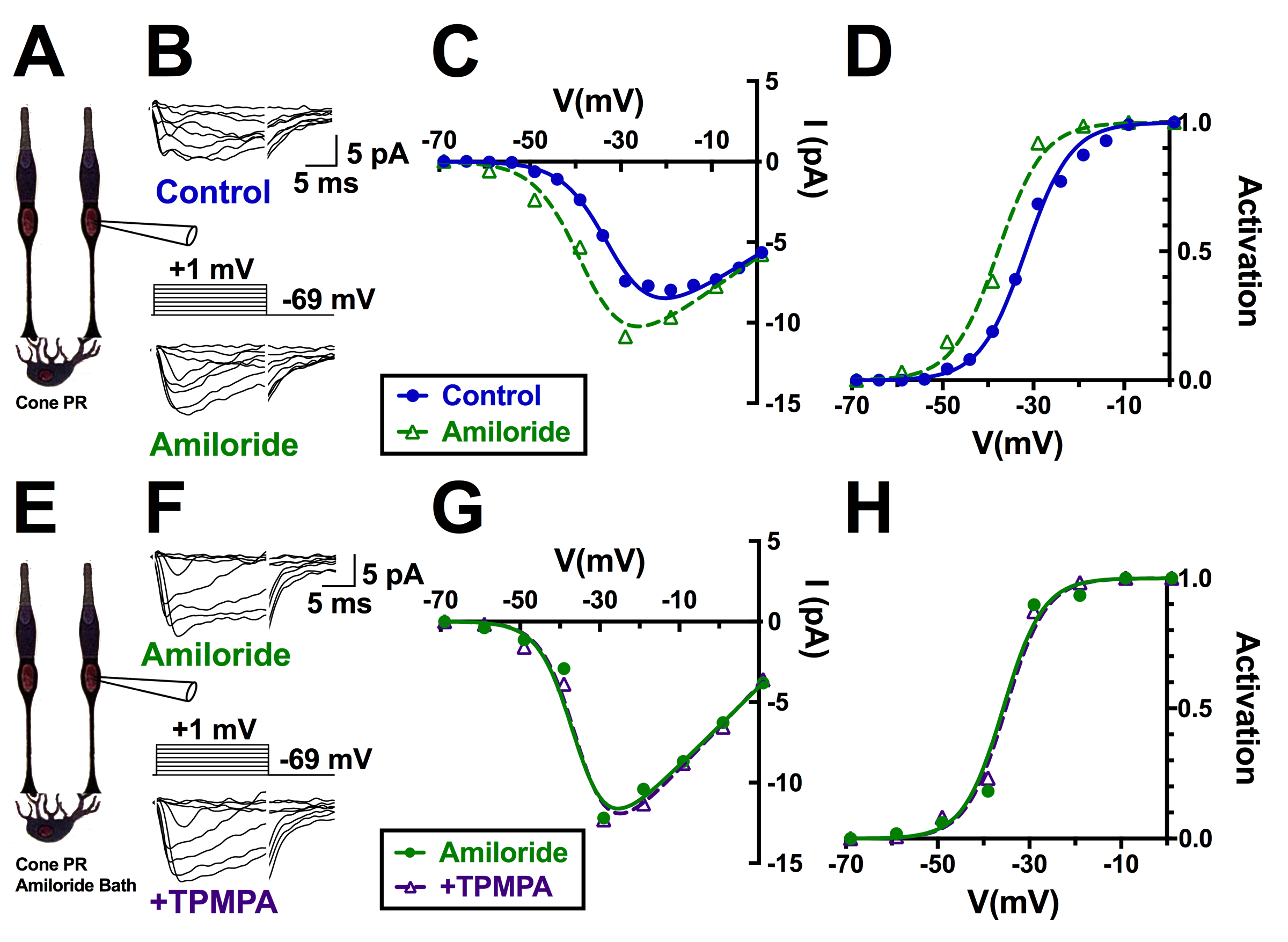

Supplement: S3 Fig — A. Patch clamp recording of a mouse cone. B. Currents elicited by the voltage steps shown in the absence (top) and presence (bottom) of the NHE-blocker amiloride (30 μM). C. I–V relations show larger CaV channel currents, activating at more negative voltages, in the presence of amiloride. D. Shift of the activation curve of the cell in (B) to more negative potential during amiloride application. E–H. Same paradigm as the experiment in A–D but retinal slice pretreated (30 min) and bathed continuously with 10 μM amiloride. Under these conditions, TPMPA fails to shift CaV channel activation curve to more negative potentials (H). Underlying data of cells in this figure can be found in S1 Data. CaV channel, voltage-gated Ca2+ channel; I–V, current–voltage; NHE, Na+/H+ exchanger; TPMPA, (1,2,5,6-tetrahydropyridin-4-yl)methylphosphinic acid. (TIFF) [file pbio.3000200.s003.tiff]

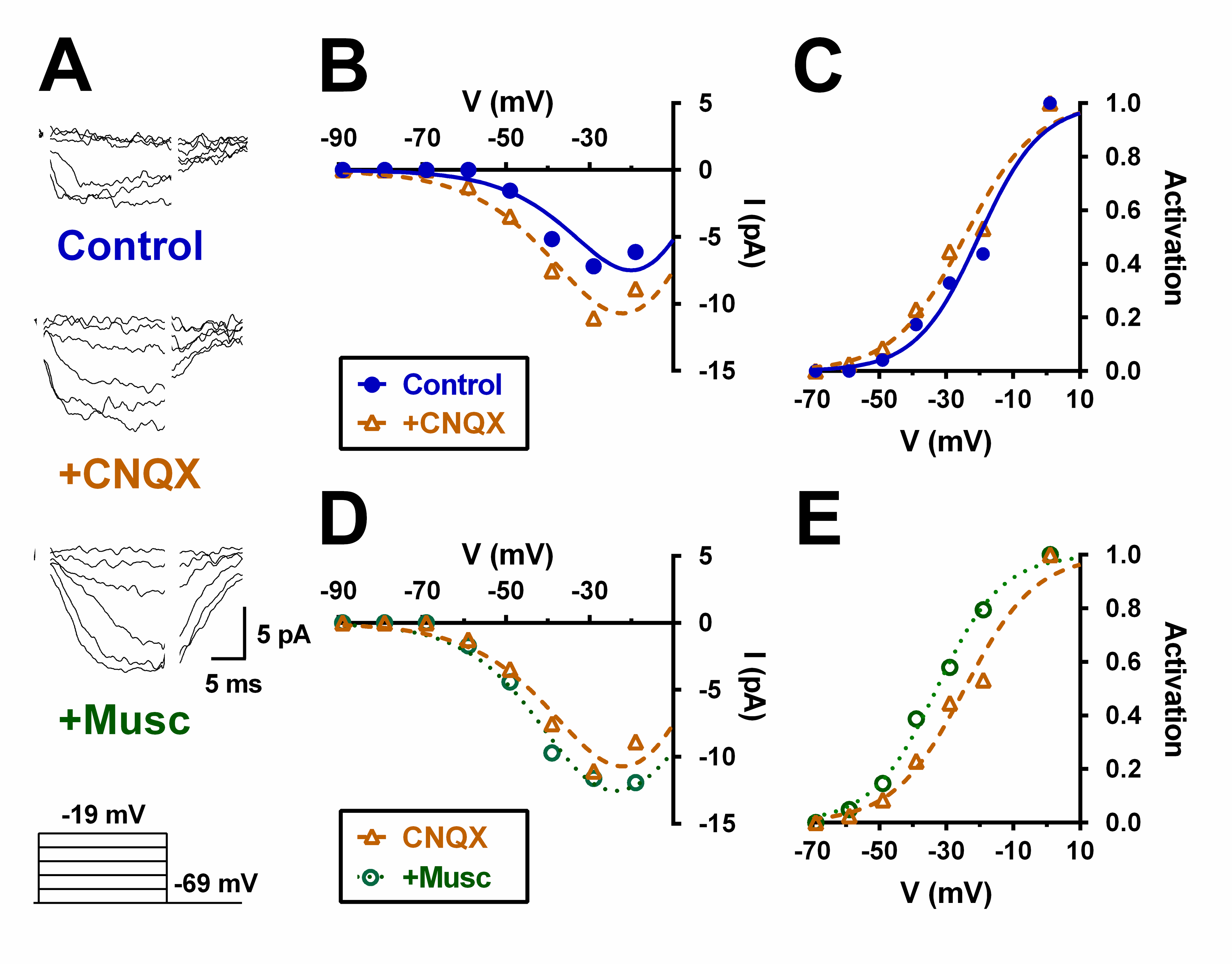

Supplement: S4 Fig — A. Currents elicited by voltage steps shown in a cone before (top) and during (middle) 50 μM CNQX application and in both 100 μM muscimol and 50 μmM CNQX (bottom). B. I–V relations show larger CaV channel currents in the presence of CNQX. C. The cone CaV channel activation curve shifts to a more negative potential during CNQX application (−20.3 mV to −24.5 mV). D. I–V relations show even larger CaV channel currents in the presence of CNQX and muscimol. E. The cone CaV channel activation curve shifts to a more negative potential during muscimol application in a cone bathed in CNQX (−24.5 mV to −31.9 mV). Underlying data of cells in this figure can be found in S1 Data. CaV channel, voltage-gated Ca2+ channel; CNQX, 6-cyano-7-nitroquinoxaline-2,3-dione; I–V, current–voltage. (TIF) [file pbio.3000200.s004.tif]

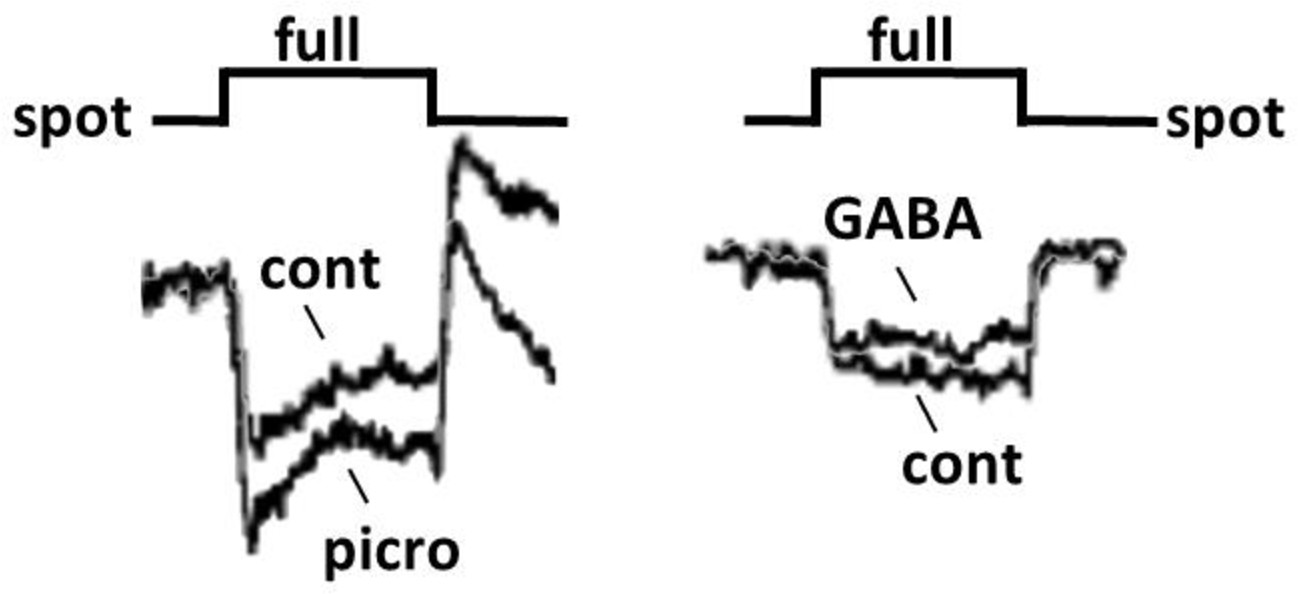

Supplement: S5 Fig — Macaque cones, voltage clamped near −40 mV, respond with an inward current when full field illumination (“full;” 0.5 s) was added to continuous spot illumination (“spot”). The control current increase (“cont”) was attributed to an increase in CaV channel and Cl(Ca) currents. In this figure, superfusion with picrotoxin (200 μM) made the inward current larger (left, “picro”) and GABA (500 μM) made the current response smaller (right), similar to the relative CaV channel current amplitude changes recorded under voltage clamp at −40 mV in cones from mouse and guinea pig during picrotoxin and muscimol superfusion in the present report. The responses to picrotoxin and GABA in macaque cones are not easily explained as being due to the presence of GABARs on cones but are what would be predicted were GABARs on horizontal cells producing changes in cleft pH to alter CaV channel activation as described in the present report. CaV channel, voltage-gated Ca2+ channel; Cl(Ca), Ca2+-activated chloride channel; GABAR, GABA receptor. (TIF) [file pbio.3000200.s005.tif]

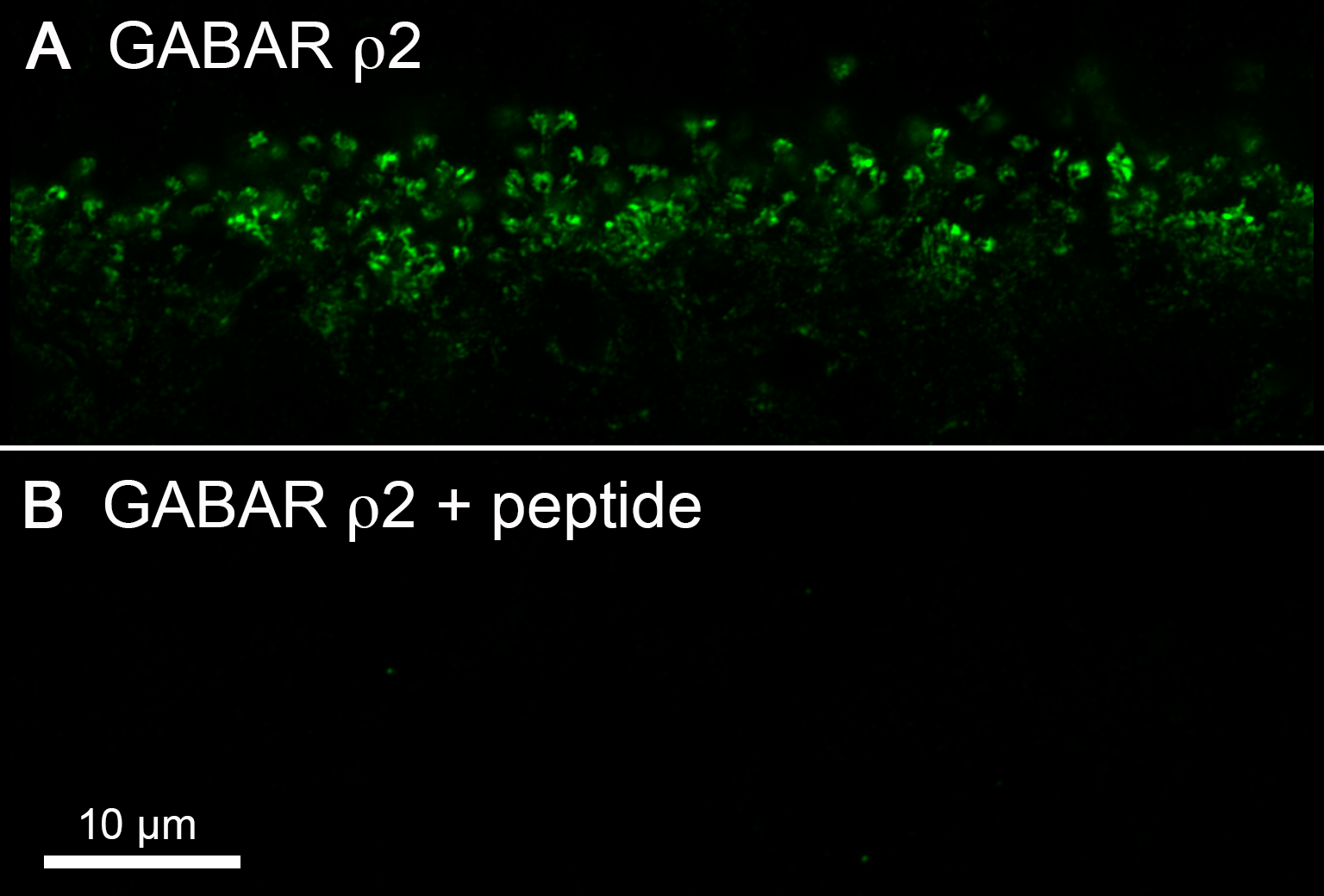

Supplement: S6 Fig — (A) GABAR ρ2 immunolabeling in the OPL of mouse retina with 1:1,000 dilution of the ρ2 antibody (AGA-007, Alomone, Jerusalem, Israel). (B) Immunolabeling by GABAR ρ2 antibody (1:1,000) preincubated with the 10−5 to 10−7 M (shown 10−6 M) antigenic peptide ([C]RKRWTGHLETSKPSH, amino acid residues 51–65 of rat GABAR ρ2, accession P47742) for 3.5 hours at 4 °C showed no specific immunoreactivity. Images obtained on a Zeiss LSM 880 with a Plan-Apochromat 63x / 1.4 Oil objective. Projection of 11 optical sections of 0.4 μm, z-step = 0.10 μm, Airyscan processed. Scale bar = 10 μm. GABAR, GABA receptor; LSM, laser scanning microscope; OPL, outer plexiform layer. (TIF) [file pbio.3000200.s006.tif]
