# Supplementary material for: Novel hybrid action of GABA mediates inhibitory feedback in the mammalian retina
Source: PLoS Biol. 2019 Apr 1;17(4):e3000200. doi: 10.1371/journal.pbio.3000200 (PMC6459543; doi:10.1371/journal.pbio.3000200)
Supplement: S1 Table — The species stated were used in electrophysiological experiments in cell types listed for the listed figure. In all immunohistochemical investigations, mouse retina was used. (DOCX) [file pbio.3000200.s007.docx]

S1 Table

| **Figure Number** | **Experimental Test** | **Cell type** | **Species** |
| --- | --- | --- | --- |
| Fig 1 | Picrotoxin, HEPES | Cones | Mouse, Rat, Guinea pig |
| Fig 2 | TPMPA, Gabazine, Strychnine | Cones | Guinea pig |
| Fig 3 | TPMPA, VGAT KO | Cones, HCs | Mouse |
| Fig 4 | PSEM | Cones, HCs | Mouse |
| Fig 5 | Muscimol, bumetanide | Cones, HCs | Mouse |
| Fig 6 | Cariporide, TPMPA | Cones | Mouse |
| Fig 7 | Muscimol, CNQX | Cones | Guinea pig |
| S2 Fig | PSEM | HC | Mouse |
| S3 Fig | Amiloride, TPMPA | Cones | Mouse |
| S4 Fig | Muscimol, CNQX | Cones | Mouse |
