# Supplementary material for: Novel hybrid action of GABA mediates inhibitory feedback in the mammalian retina
Source: PLoS Biol. 2019 Apr 1;17(4):e3000200. doi: 10.1371/journal.pbio.3000200 (PMC6459543; doi:10.1371/journal.pbio.3000200)
Supplement: S2 Table — Picrotoxin did not cause a change in the resting cone conductance measured between −90 and −60 mV in rats, mice, or guinea pigs. The same result (no change in conductance) was obtained in rats when Hepes was present in the bath. Muscimol, TPMPA, gabazine, and strychnine tested on guinea pig did not change cone conductance. TPMPA did not produce a change in the resting cone conductance measured mice. The slope conductance of lines fit to the I–V relation of each cone between −90 and −60 mV were compared with F-tests and adjusted R2. I–V, current–voltage; TPMPA, (1,2,5,6-tetrahydropyridin-4-yl)methylphosphinic acid. (DOCX) [file pbio.3000200.s008.docx]

S2 Table

| **Drug** | **Species** | **ΔG (-90 to -60 mV)** | **P value** | **F-test, R2** | **N** |
| --- | --- | --- | --- | --- | --- |
| Picrotoxin | Rat | none | 0.9035 | -0.07566 | 5 |
| Picrotoxin | Mouse | none | 0.7611 | -0.06899 | 5 |
| Picrotoxin | Guinea pig | none | 0.1459 | 0.06101 | 7 |
| Picrotoxin & Hepes | Rat | none | 0.5703 | -.04954 | 5 |
| Muscimol | Guinea pig | none | 0.6645 | -0.04199 | 7 |
| TPMPA | Guinea pig | none | 0.6813 | -0.0625 | 5 |
| TPMPA | Mouse | none | 0.4355 | -0.02165 | 6 |
| Gabazine | Guinea pig | none | 0.8426 | -0.08426 | 7 |
| Strychnine | Guinea pig | none | 0.9725 | -0.06242 | 6 |
